# Expression pattern of resynthesized allotetraploid *Capsella* is determined by hybridization, not whole‐genome duplication

**DOI:** 10.1111/nph.18542

**Published:** 2022-11-07

**Authors:** Tianlin Duan, Adrien Sicard, Sylvain Glémin, Martin Lascoux

**Affiliations:** ^1^ Department of Ecology and Genetics, Evolutionary Biology Centre and Science for Life Laboratory Uppsala University 75236 Uppsala Sweden; ^2^ Department of Plant Biology Swedish University of Agricultural Sciences 750 07 Uppsala Sweden; ^3^ UMR CNRS 6553 ECOBIO Campus Beaulieu, bât 14a, p.118, CS 74205 35042 Rennes France

**Keywords:** *Capsella bursa‐pastoris*, gene expression, hybridization, neopolyploid lines, polyploidy

## Abstract

- Polyploidization, the process leading to the increase in chromosome sets, is a major evolutionary transition in plants. Whole‐genome duplication (WGD) within the same species gives rise to autopolyploids, whereas allopolyploids result from a compound process with two distinct components: WGD and interspecific hybridization.
- To dissect the instant effects of WGD and hybridization on gene expression and phenotype, we created a series of synthetic hybrid and polyploid *Capsella* plants, including diploid hybrids, autotetraploids of both parental species, and two kinds of resynthesized allotetraploids with different orders of WGD and hybridization.
- Hybridization played a major role in shaping the relative expression pattern of the neo‐allopolyploids, whereas WGD had almost no immediate effect on relative gene expression pattern but, nonetheless, still affected phenotypes. No transposable element‐mediated genomic shock scenario was observed in either neo‐hybrids or neo‐polyploids. Finally, WGD and hybridization interacted and the distorting effects of WGD were less strong in hybrids. Whole‐genome duplication may even improve hybrid fertility.
- In summary, while the initial relative gene expression pattern in neo‐allotetraploids was almost entirely determined by hybridization, WGD only had trivial effects on relative expression patterns, both processes interacted and had a strong impact on physical attributes and meiotic behaviors.

## Introduction

Polyploidization, the process leading to the increase in chromosome sets, is frequent in plants, with *c*. 24% of extant plant species being polyploids (Barker *et al*., 2016), and numerous ancient polyploidization events have been discovered in ferns and angiosperms (Wood *et al*., 2009; Wendel, 2015; Zhang *et al*., 2020). Whole‐genome duplication (WGD) within the same species gives rise to autopolyploids, whereas allopolyploids result from a compound process with two distinct components: an increase in the number of chromosome sets and interspecific hybridization. The two components differ in mechanisms, and each alone can cause profound changes in genome functioning and phenotype of an organism.

The sudden change in ploidy level can be challenging during early stages. First, the duplicated genome size instantly increases DNA content and cell size (Beaulieu *et al*., 2008; Allario *et al*., 2011; Yao *et al*., 2011). This physical alteration has a series of nucleotypic effects (Doyle & Coate, 2019), including changes in surface : volume ratio, concentrations of gene products, and cell cycle length (Šímová & Herben, 2012). Dosage‐sensitive genes may be disproportionately affected, thus creating an imbalance between essential cellular components. Together, these DNA content‐induced nucleotypic effects can already explain many typical polyploid phenotypes, such as larger stomata, enlarged organs, and different growth rates (Miller *et al*., 2012; Xu *et al*., 2020). While some polyploid phenotypes can be beneficial, many disturb physiological processes or entail larger energy and nutrient costs (Cavalier‐Smith, 2005; Veselý *et al*., 2013; Guignard *et al*., 2016; Anneberg & Segraves, 2020) and thus are potential burdens to an organism. Another serious challenge of polyploidy occurs during meiosis. With more than two sets of homologous chromosomes and low differentiation, neo‐autopolyploids often form multivalents and entanglements during meiotic synapsis, leading to mis‐segregation and reduced fertility (Yant *et al*., 2013; Bomblies *et al*., 2016).

Like WGD, interspecific hybridization is pervasive in plants (Whitney *et al*., 2010). The union of divergent genomes itself is an influential change, shifting the entire gene expression pattern toward midparent values. More importantly, newly formed hybrids also face the immediate deleterious effects caused by the incompatibility between divergent genomes. Several mechanisms can generate incompatibilities. First, gene regulatory networks have evolved independently in each parental lineage, accelerated by both organism‐level natural selection (McGirr & Martin, 2020) and compensatory coevolution of regulatory elements (Landry *et al*., 2005; Fyon *et al*., 2015). Consequently, when the diverged regulatory networks are combined through hybridization, intergenomic interactions between regulatory elements can impair hybrid fitness through the dysregulation of gene expression (Maheshwari & Barbash, 2012; Hu & Wendel, 2019). Second, merging diverged genomes can further upset the genomic stochiometric balance in hybrids. One special form of stoichiometric imbalance occurs between maternally and paternally expressed genes in the endosperm of hybrids, which causes hybrid seed failure in *Solanum* (Roth *et al*., 2019), *Mimulus* (Coughlan *et al*., 2020), and *Capsella* (Lafon‐Placette *et al*., 2018). Third, diploid hybrids suffer from meiotic abnormalities such as autopolyploids, but the mechanism is different. In diploid hybrids, there are no more homologous chromosome pairs but only homeologous ones. The low affinity between the diverged homeologous chromosomes can lead to chromosome asynapsis and various abnormalities in gametes, including chromosome damage, aneuploids, and unreduced gametes (Szadkowski *et al*., 2010; Mason *et al*., 2011). Irregular chromosome pairing during meiosis also explains hybrid sterility in many taxa (Kamstra *et al*., 1999; Bhattacharyya *et al*., 2013; Torgasheva & Borodin, 2016; Liang & Sharakhov, 2019). In addition, the mismatch between divergent transposable elements (TEs) and TE‐suppressors (Parisod *et al*., 2010; Hénault, 2021) and nuclear‐cytoplasmic conflicts (Bogdanova & Galieva, 2009) can also generate expression novelties and affect fitness in hybrids.

Both WGD and hybridization are expected to bring instant stress to an organism, but the relative impact of WGD and hybridization on allopolyploid organisms is less clear since the two processes have seldom been quantified separately in the same system. This also prevented rigorous identification of the real initial contributors to major allopolyploidization consequences, such as the breakdown of self‐incompatibility (Mable, 2004; Husband *et al*., 2008; Novikova *et al*., 2017; Zenil‐Ferguson *et al*., 2019), or the genomic remodeling due to TE‐reactivation, also known as ‘genomic shock’ (McClintock, 1984). The expression ‘genomic shock’ was initially coined to describe the *stochastic* genomic restructuring caused by an instant reactivation of TEs under several forms of stress, including hybridization (McClintock, 1984). As a large number of subsequent studies on genomic shock were conducted in allopolyploid species (reviewed in Vicient & Casacuberta, 2017), sometimes, genomic shock was also assumed to be an outcome of WGD (Bardil *et al*., 2015; Fasano *et al*., 2016; Baduel *et al*., 2018). Later, the concept of shock was also extended to include more forms of drastic genomic changes, regardless of the underlying mechanism (Hegarty *et al*., 2006; Buggs *et al*., 2011; Zhang *et al*., 2016). In this study, we used a narrow concept of ‘genomic shock’ (McClintock, 1984; Baduel *et al*., 2018), ‘TE‐mediated genomic shock’, in which hybridization or WGD function as global genomic stress and immediately cause the reactivation and proliferation of previously epigenetically silenced TEs across the genome. Both the transcriptional activation and transposition of TEs could drastically affect nearby gene expression (Hirsch & Springer, 2017). Therefore, a TE‐mediated genomic shock triggered by hybridization or WGD is one possible cause of nonadditive gene expression (expression levels deviate from midparent values) in allopolyploids.

Potentially important is the interaction between WGD and hybridization. Either WGD or hybridization alone can bring huge shifts to a genome and be overwhelming for an organism. Yet, the combination of these two processes, allopolyploidy, is surprisingly common in plants, and *c*. 11% of plant species are allopolyploids (Barker *et al*., 2016). Despite a potential underestimation of the WGD (Soltis *et al*., 2007) or hybridization rate (Taylor & Larson, 2019; Suvorov *et al*., 2020), the high fraction of allopolyploid species suggests beneficial interactions between WGD and hybridization. Several short‐term interactions may explain the abundance of allopolyploid species. First, during the formation of allopolyploids, hybridization can directly trigger polyploidization, as the lack of chromosome pairing in meiosis I increases the rate of unreduced gametes (Ramsey & Schemske, 1998; Mason *et al*., 2011). Second, both diploid hybrids and autopolyploids suffer from different meiotic problems, but theoretically, both problems may be mitigated by combining the two processes. Interspecific hybridization brings homeologous chromosomes to autopolyploids. Sequence divergence between the chromosomes of different species limits multivalent formation and facilitates chromosome segregation. On the contrary, polyploidization restores chromosome pairing during meiosis in hybrids and reduces the independent assortment of chromosomes in diploid hybrids, therefore helping to retain the original genomic stochiometric balance among chromosome pairs. In allopolyploids, the interactions between hybridization and WGD were mixed with the main effects of the two processes. More experimental work incorporating both WGD and hybridization as factors would help separate the different components and evaluate their relative contributions.

The genus *Capsella* is a good model system for studying allopolyploidy. Two diploid species, *C. orientalis* Klokov and *C. grandiflora* (Fauché & Chaub.) Boiss., split *c*. 900 000 yr ago. Then, 100 000–300 000 years ago (Douglas *et al*., 2015), the hybridization of ancestral populations of these two lineages yielded an allotetraploid species, *C. bursa‐pastoris* (L.) Medik. The maternal progenitor of *C. bursa‐pastoris* is the *C. orientalis* lineage (Hurka *et al*., 2012). *Capsella grandiflora* is self‐incompatible, whereas *C. orientalis* and *C. bursa‐pastoris* are self‐compatible and predominantly selfing. *Capsella bursa‐pastoris* has been a successful allopolyploid species with a world‐wide distribution (Hurka *et al*., 2012).

In this study, we created a unique series of synthetic hybrid and polyploid *Capsella* plants to dissect the instant effects of WGD and hybridization and their interaction on gene expression and phenotypes. Specifically, we addressed three questions: (1) what are the relative impacts of WGD and hybridization on gene expression patterns and phenotypes of neo‐allopolyploids? (2) Is there a TE‐mediated genome shock after WGD or hybridization? And (3) can we identify any instant interaction between WGD and hybridization?

## Materials and Methods

### Generating diploid hybrids, autotetraploids, and allotetraploids

*Capsella bursa‐pastoris‐like* allotetraploids were resynthesized with the diploid *C. orientalis* and *C. grandiflora*, which are the closest extant relatives of the progenitors of the natural *C. bursa‐pastoris*, and likely retained the same mating system as the real progenitors. To control for within‐group genetic variation, one inbred line of *C. orientalis* (ID: URAL‐RUS5; collected at 55.11N, 61.39E) and seeds from two wild individuals from the same *C*. *grandiflora* population (ID: 81; collected at 37.30N, 22.06E) were used for resynthesizing allotetraploids.

**Fig. 1:**
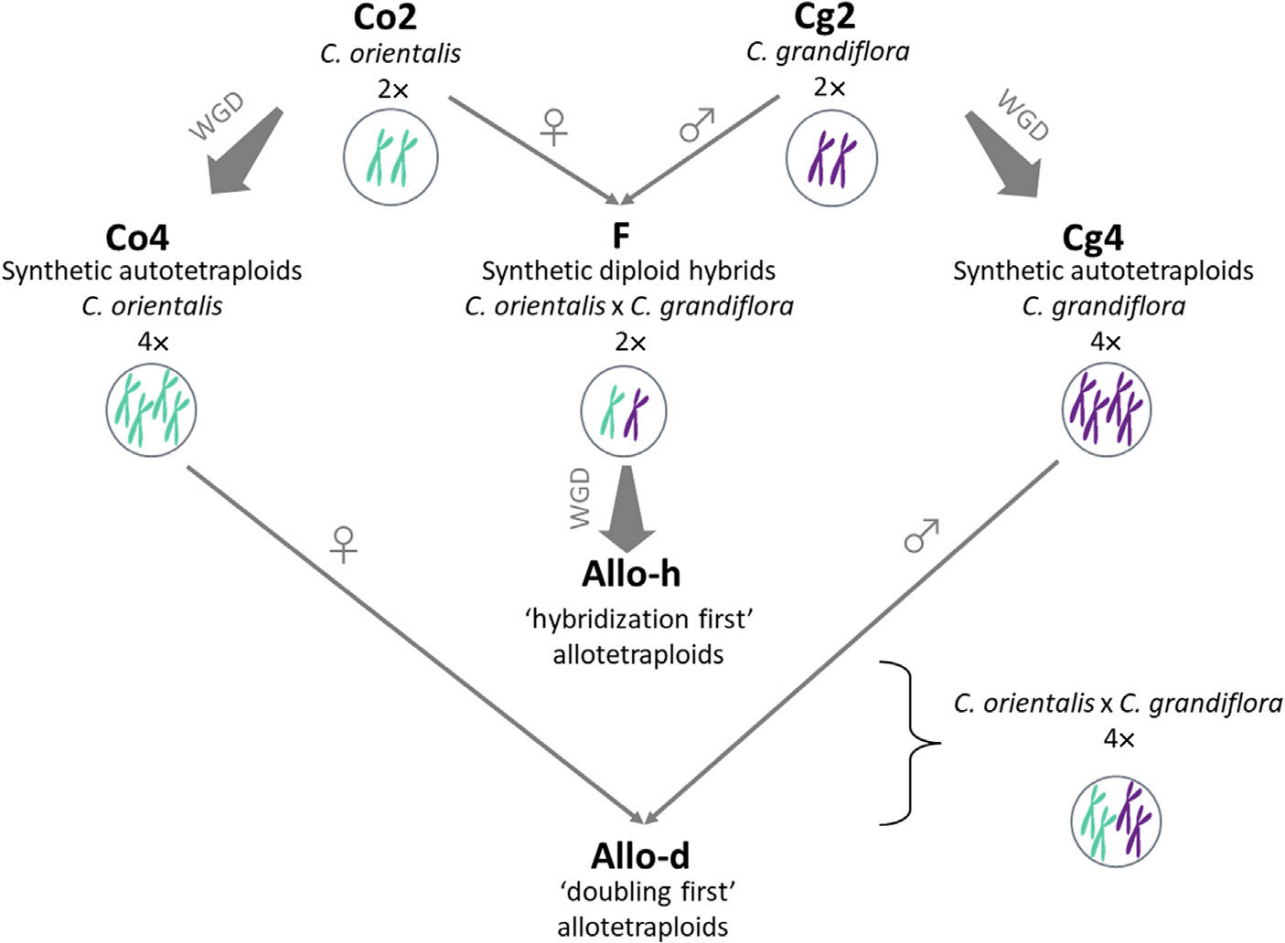
The even groups of synthetic or natural *Capsella* plants used in this study. Synthetic diploid hybrids, autotetraploids, and allotetraploids were generated with the diploid *Capsella orientalis* and *Capsella grandiflora*. Whole‐genome duplication (WGD) was induced by colchicine treatments. *Capsella orientalis* served as maternal plant in all interspecific crosses. Group abbreviations are highlighted in bold.

Autotetraploid *C. orientalis* and *C. grandiflora*, and tetraploid hybrids were generated by treating the diploid seedlings with 0.25% (w/v) colchicine solution. Hybrids were generated by pollinating *C. orientalis* with *C. grandiflora* pollen to mimic the formation of the natural allotetraploid species, *C. bursa‐pastoris* (Hurka *et al*., 2012). Since no interspecific crosses yielded viable seeds in test trials, all interspecific hybrids were generated through ovule rescue (Monnier, 1984; Supporting Information Methods S1). The allotetraploid plants were resynthesized in two ways: the ‘hybridization‐first’ allotetraploids (Allo‐h) were obtained by treating the F1 diploid hybrids with colchicine solution, whereas the ‘doubling‐first’ (Allo‐d) allotetraploids were obtained by crossing the autotetraploid *C. orientalis* and *C. grandiflora* (Fig. 1). The ploidy level of all the synthetic tetraploid plants was verified with flow cytometry analysis on cauline leaves, and the hybrid identity of all the diploid and tetraploid interspecies hybrids was confirmed using a PCR‐based marker (Table S1).

To compare the plants' phenotypes and gene expression patterns, the synthetic diploid hybrids, autotetraploids, and allotetraploids were grown together in a growth chamber, along with the two diploid parental species, resulting in seven groups of plants (Fig. 1). The second generation of synthetic diploid hybrids, autotetraploids, and allotetraploids was used in this comparison, so that the influence from ovule rescue and colchicine treatment was mitigated.

### Phenotyping

Each of the seven plant groups was represented by six randomly chosen ‘lines’. For the two diploid parental species, ‘lines’ are equivalent to individuals from previous generations (families). Whereas for the five synthetic groups, each line represented an independent hybridization event (an initially rescued embryo) or different polyploidization events, except for the Co4 group, in which the six lines were from three polyploidization events. For each line, six individuals were grown as replicates. The six individuals were full siblings from brother–sister mating (Cg2 and Cg4 groups) or self‐fertilization of the parental plants (the other five groups). In total, 252 plants (7 groups × 6 lines × 6 individuals) were grown for measuring phenotypes. For detailed growing conditions, see Methods S2.

The number of pollen grains per flower and pollen viability were calculated for 215 plants by counting suspended pollen on a hemocytometer (Methods S3; Fig. S1a). Pollen viability was evaluated by an aceto‐carmine staining method (Methods S3), modified from Marks (1954).

The ability to produce seeds with only autonomous selfing was recorded as a categorical trait. The length of the longest branch was measured on dry plants, as a vegetative growth trait. The 11^th^–20^th^ fruits from the main stem were collected separately for measuring the ratio of normal and abnormal seeds. Seeds that were flat or small and dark were considered abnormal (Fig. S1b). If only partial flowers set fruits, then the first 10 fruits were collected, regardless of their positions. Individuals with < 10 fruits were excluded from the analysis.

### Sampling for RNA‐sequencing

Leaves and inflorescences samples were collected from all individuals included in the phenotyping experiment, but for each line, only one randomly chosen individual was retained for RNA‐sequencing, summing up to 84 samples (7 groups × 6 lines × 2 tissues).

For leaf samples, the eighth and ninth leaves were harvested at the emergence of the 11^th^ leaf. For inflorescence samples (hereinafter called ‘flower samples’), 7–14 d after the first flower opened, two to four inflorescences with only unopened flower buds were collected from each plant. The samples were collected between 16:00 and 18:00 h. The collected samples were snap‐frozen in liquid nitrogen and stored at −80°C until RNA extraction.

### RNA‐sequencing and data preparation

Total RNA was extracted from leaves and inflorescences with a cetyl‐trimethyl‐ammonium‐bromide‐based method (Methods S4), modified from Chang *et al*. (1993). Sequencing libraries were prepared by the SNP&SEQ Technology Platform in Uppsala, using the Illumina TruSeq Stranded mRNA (poly‐A selection) kit, and sequenced on three NovaSeq 6000 S4 lanes, with pair‐end reads of 150 bp. One library was prepared for each diploid sample, and two libraries were prepared for each tetraploid sample. The RNA‐sequencing reads of the same individual were later combined. All RNA‐sequencing data are stored on SRA BioProject PRJNA848625.

The quality of the raw RNA‐sequencing reads (Fastq format) was inspected with FastQC (Andrews, 2010) and MultiQC (Ewels *et al*., 2016). The RNA‐sequencing reads were then mapped to the reference genome of *C*. *rubella* (Slotte *et al*., 2013) with Stampy v.1.0.32 (Lunter & Goodson, 2011). For mapping, the expected divergence from the reference (‐‐substitutionrate) was set to 0.02, 0.04, and 0.025 for *C. grandiflora*, *C. orientalis*, and hybrids, respectively. We used the same substitution rate for tetraploids and the corresponding diploids. SAMtools v.1.5 (Danecek *et al*., 2021) was used for format conversion between steps. The mapping quality was then checked by Qualimap v.2.2.1 (Okonechnikov *et al*., 2016). The number of reads mapped to each transcript (total gene expression of both homeologs) was determined by HTSeq v.0.12.4 (Anders *et al*., 2015). Only the reads that were unambiguously mapped to a single transcript were counted (mode ‘union’ in HTSeq).

To reduce the effect of differences in sequencing depths, we downsampled the mapped reads with a custom Python script, so that the seven groups had a similar average number of mapped reads (Methods S5).

### Transcriptome‐wide expression pattern

The transcriptome‐wide expression pattern was viewed by using a multidimensional scaling (MDS) plot with the R package edgeR (v.3.28.1; Robinson *et al*., 2010), using genes with transcripts‐per‐million (TPM, Wagner *et al*., 2012) > 2 in at least three samples. The gene expression levels were normalized with the trimmed mean of *M*‐values (TMM) method.

As the samples were primarily grouped by tissues, two separate MDS plots were then made for flowers and leaves, respectively, using the same expression filter and normalization method. Results of MDS analysis were verified with principal component analyses, using TMM‐normalized TPM values of the same set of genes (Fig. S3).

### Differential expression analysis

Differential expression (DE) analyses were performed separately on flower or leaf samples using the edger package. The gene expression levels were normalized with the TMM method. A single model (~0 + group indicator) was fitted to the samples of all seven groups, followed by pair‐wise contrasts. The DE was tested on genes of which the fold‐changes (FCs) were > 2, and genes with a false discovery rate (FDR) < 0.05 were considered as significantly differentially expressed genes (DEGs).

### Identifying nonadditive gene expression

For diploid hybrids and allotetraploids, both interactions between diverged regulatory elements and a TE‐mediated genomic shock are expected to cause nonadditive gene expression, the deviation from midparent expression levels. We further classified gene expression in diploid/tetraploid hybrids into 10 additive or nonadditive expression categories (Table S2) by comparing gene expression levels in hybrids and both diploid parental species, using the results of DE analyses (FC > 2 and FDR < 0.05). The 10 expression categories were modified from Zhang *et al*. (2016). We focused on complete expression level dominance (ELD, the expression level in hybrids is similar to that in one of the parental species but not the other) and transgressive gene expression (TRE, the expression level in hybrids is higher or lower than in both parental species). Genes with TRE or complete ELD were considered as proven nonadditive gene expressions. The recognized nonadditively‐expressed genes were compared among the three hybrid groups and with parental expression‐differential (PED) genes.

### Extreme gene expression

As a part of a TE‐mediated genomic shock scenario, new TE transpositions can also cause individual‐specific drastic gene expression changes. However, the DE analyses can only identify group‐level gene expression changes, and individual‐specific expression changes were omitted. To supplement the group‐level DE analysis, we also compared the extent of dysregulation at the individual level by counting the number of extremely expressed genes in each individual. If the TE‐mediated genomic shock has large effects on expression patterns, we would expect to see the hybrids or polyploids possessing more extremely expressed genes than the parental species. Genes with CPM > 1 in at least two individuals were used in this analysis. The 42 individuals (7 groups × 6 individuals) were ranked for each gene by the CPM normalized gene expression levels. Ranks 1 and 42 were regarded as ‘extreme expression’. To mitigate the effect of midparent expression in hybrids, the same analysis was repeated with only the genes that were not differentially expressed between the Cg2 and Co2 groups (FC < 1.2 or FDR > 0.05).

### TE abundance

To test whether a TE‐mediated genomic shock occurred in the newly generated hybrids or polyploids, we compared the TE abundance in transcriptomes among the seven plant groups. If there is an ongoing large‐scale TE‐reactivation and proliferation in hybrids or polyploids, we expect both autonomous and passive TE transcription to increase and, therefore, a higher TE abundance in transcriptomes. Repeat sequences were first identified and annotated in the *C. rubella* reference genome (Slotte *et al*., 2013) with the software repeatmasker v.4.1.0 (Smit *et al*., 2015), using Brassicaceae repeat sequences from Dfam_3.1 (Storer *et al*., 2021) and RepBase‐20181026 (Bao *et al*., 2015) as the reference repeat database. Simple repeats were excluded by the ‘‐nolow’ option. Then, the number of reads mapped to genes and TEs was quantified simultaneously with the tecount package v.2.2.1 (Jin *et al*., 2015). The total number of reads that mapped to genes and TEs was downsampled as before (Methods S5), so that the seven plant groups have a similar average library size. Finally, the proportions of annotated reads mapped to TEs in each RNA‐sequencing sample (number of reads mapped to TEs/number of reads mapped to TEs or genes) were compared among the seven plant groups. Aside from the overall expression level of TEs, we also explored whether the expression level of some major TE categories was significantly increased by hybridization or WGD, as reported in previous studies (Vicient & Casacuberta, 2017). The proportion of reads that were mapped to long terminal repeat (LTR), helitron, short interspersed nuclear elements (SINEs), and long interspersed nuclear elements (LINEs) was compared among the seven groups.

### Statistical analysis

The difference in stem length, flowering time, number of pollen grains per flower, and extremely expressed genes per individual among the seven plant groups was tested by one‐way analysis of variance (ANOVA) and Tukey's HSD test (R package ‘agricolae’, de Mendiburu & Yaseen, 2020).

To test the effect of WGD and hybridization on the ratio of viable : nonvariable pollen grains, normal : abnormal seeds, or reads mapped to TEs : genes, generalized linear models (GLMs) were fitted to the data in the R software environment v.3.6.3 (R Core Team, 2020), using both ploidy level (2×/4×) and hybrid state (hybrid/nonhybrid) as categorical factors. For all GLMs, a quasi‐binomial error distribution with a logit link function was used to account for overdispersion. The effect of the factors and their interaction were then tested with *F*‐tests using the car R package (Fox & Weisberg, 2019). For the analyses on TE abundance, the sequencing batch/lane identity was also added to the GLMs.

## Results

### Hybridization and WGD affect flowering phenology

**Fig. 2:**
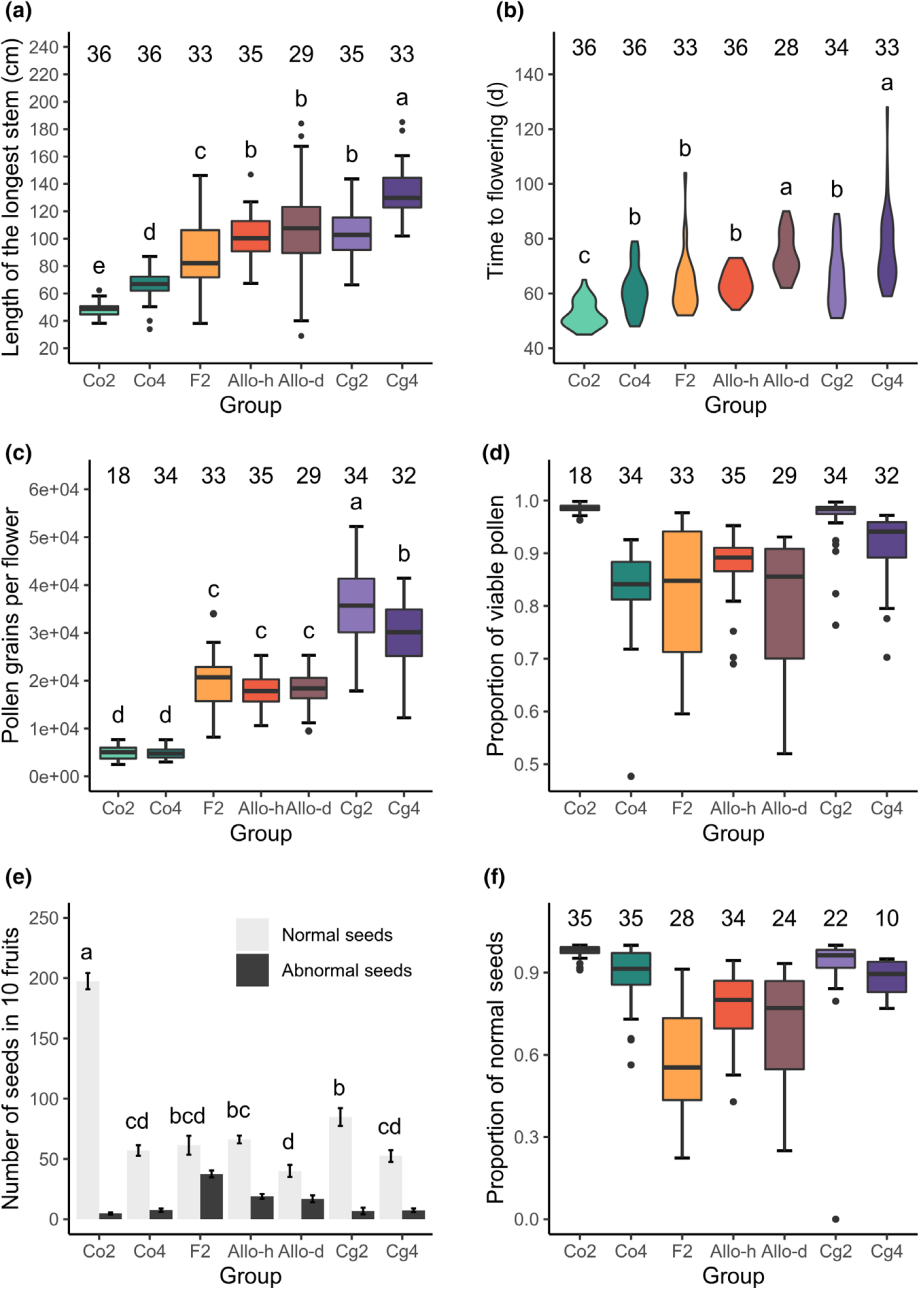
Phenotypes of the seven plant groups. (a) Stem length. (b) Flowering time. (c) Estimated pollen grain number per flower, averaged between the two flowers of each individual. (d) The proportion of viable pollen grains, calculated by examining > 300 pollen grains per flower, and averaged between two flowers. (e) The number of normal and abnormal seeds in 10 fruits. Error bars show the group mean ± SE. (f) The proportion of normal seeds (normal seeds/total seeds). Individuals with < 10 fruits were excluded from the analysis. For boxplots and violin plots, sample size (number of examined individuals) is shown above the groups. When one‐way ANOVA and Tukey's HSD test were applied (plots a–c and number of normal seeds in plot e), group difference at a significance level of α < 0.05 is indicated by letters. Groups with the same letter are not significantly different. Within each box plot, the bold horizontal line represents the median value; box range means values within interquartile range (IQR) from first quartile (Q1) to third quartile (Q3); up and down whiskers indicate 1.5 IQR above the Q3 (Q3+1.5 IQR) and 1.5 IQR below the Q1 (Q1−1.5 IQR), separately; circles representative outliers.

Both hybridization and WGD had obvious effects on stem length and flowering time, and the two traits showed similar overall patterns across the seven groups (Fig. 2a,b). The selfing *C. orientalis* and the outcrossing *C. grandiflora* represented the two extremes of the traits, while the hybrids had intermediate values between the parents of the same ploidy level. The synthetic autotetraploids had longer stems and flowered later than the corresponding diploids. In addition, the two allotetraploid groups with different orders of hybridization and WGD, Allo‐d and Allo‐h, differed in flowering time. The Allo‐h and the diploid F2 groups had similar flowering times, whereas Allo‐d plants flowered significantly later (Stem length: one‐way ANOVA, *F*_6,230_ = 66.1, *P* < 0.001; Tukey's HSD test, α = 0.05. Flowering time: one‐way ANOVA, *F*_6,229_ = 24.6, *P* < 0.001; Tukey's HSD test, α = 0.05).

### Both hybridization and WGD decrease pollen viability but are less deleterious when coupled

Pollen viability is a major fitness component and an indicator of meiotic abnormalities (Srivastava *et al*., 1992; Jiang *et al*., 2013). If there were interactions between WGD and hybridization during meiosis, the interaction would be expected to be reflected in pollen viability. We measured the number of pollen grains per flower and pollen viability in the seven groups. The selfing and outcrossing parental groups have the lowest and highest number of pollen grains, respectively, and the synthetic hybrids had intermediate values between the parental species (Fig. 2c). The synthetic tetraploids were similar to the diploids, except for the Cg4 group, which had fewer pollen grains than Cg2 (one‐way ANOVA, *F*_6,208_ = 127.7, *P* < 0.001; Tukey's HSD test, α = 0.05).

In contrast to the other traits, the two natural species, Cg2 and Co2, had the highest proportion of viable pollen grains (Cg2, 0.966 ± 0.008; Co2, 0.985 ± 0.002, Fig. 2d), and all the synthetic groups had lower proportions (F, 0.827 ± 0.020; Allo‐d, 0.813 ± 0.023; Allo‐h, 0.878 ± 0.010; Co4, 0.831 ± 0.014; Cg4, 0.914 ± 0.011). When both hybrid state (hybridization) and ploidy level (WGD) were included in the GLM, only hybridization had a main effect on the proportion of viable pollen grains, and the main effect of WGD was not significant (Table S3). There were interactions between hybridization and WGD (Table S3), indicating that the effect of WGD on pollen viability differs between hybrid and nonhybrid plants. For the nonhybrid plants, WGD decreased the proportion of viable pollen grains, whereas, among the hybrid plants, WGD did not have significant effects on pollen viability (Table S3). If only considering the ‘hybridization‐first’ allotetraploids (Allo‐h) and diploid hybrids (F), pollen viability of allotetraploids was slightly higher than the diploid hybrids (GLM, quasi‐binomial, *F*_1,66_ = 4.56, *P* = 0.0365).

The two allotetraploid groups with different orders of hybridization and WGD, Allo‐d and Allo‐h, had different proportions of viable pollen grains (GLM, quasi‐binomial, *F*_1,62_ = 6.38, *P* = 0.0141). While the Allo‐h group was slightly higher than group F, group Allo‐d was similar to F.

### Both hybridization and WGD induced abnormal seeds, but WGD increased seed quality among hybrids

The ability to produce seeds with only autonomous selfing was recorded. Synthetic autotetraploids and corresponding diploids had the same selfing ability. All Co4 plants successfully generated seeds by autonomous selfing, and all Cg4 plants failed to generate any seed without hand pollination. Both diploid and tetraploid hybrids showed a mixed pattern. With only autonomous pollination, all individuals (35) of the Allo‐h group were able to produce some seeds, but some individuals of the Allo‐d (eight of 35) or F2 group (seven of 29) failed to produce seeds.

To compare further the impact of hybridization and WGD on fitness components, we measured the proportion of normal seeds in the seven groups (Fig. 2e,f). All hybrid or tetraploid groups had fewer normal seeds per fruit than diploid parental groups (one‐way ANOVA, *F*_6,181_ = 94.9, *P* < 0.001; Tukey's HSD test, α = 0.05). Both hybridization and WGD significantly affected the proportion of normal and abnormal seeds, and the interactions between the two variables were significant (Table S3). WGD decreased the proportion of normal seeds among the nonhybrid groups but increased it for hybrid groups. The proportions of normal seeds of the F, Allo‐h, and Allo‐d groups were 0.57 ± 0.03, 0.78 ± 0.02, and 0.70 ± 0.04, respectively.

### Transcriptome‐wide expression pattern was determined by hybridization, not WGD

Gene expression was compared across the seven groups using RNA‐sequencing data from leaves and flowers. Each tissue of each group was represented by six individuals, resulting in 84 RNA‐sequencing libraries (Table S4). After mapping and read downsampling, on average, 36.9 ± 1.8 million read pairs were kept for each RNA‐sequencing sample (Fig. S2).

**Fig. 3:**
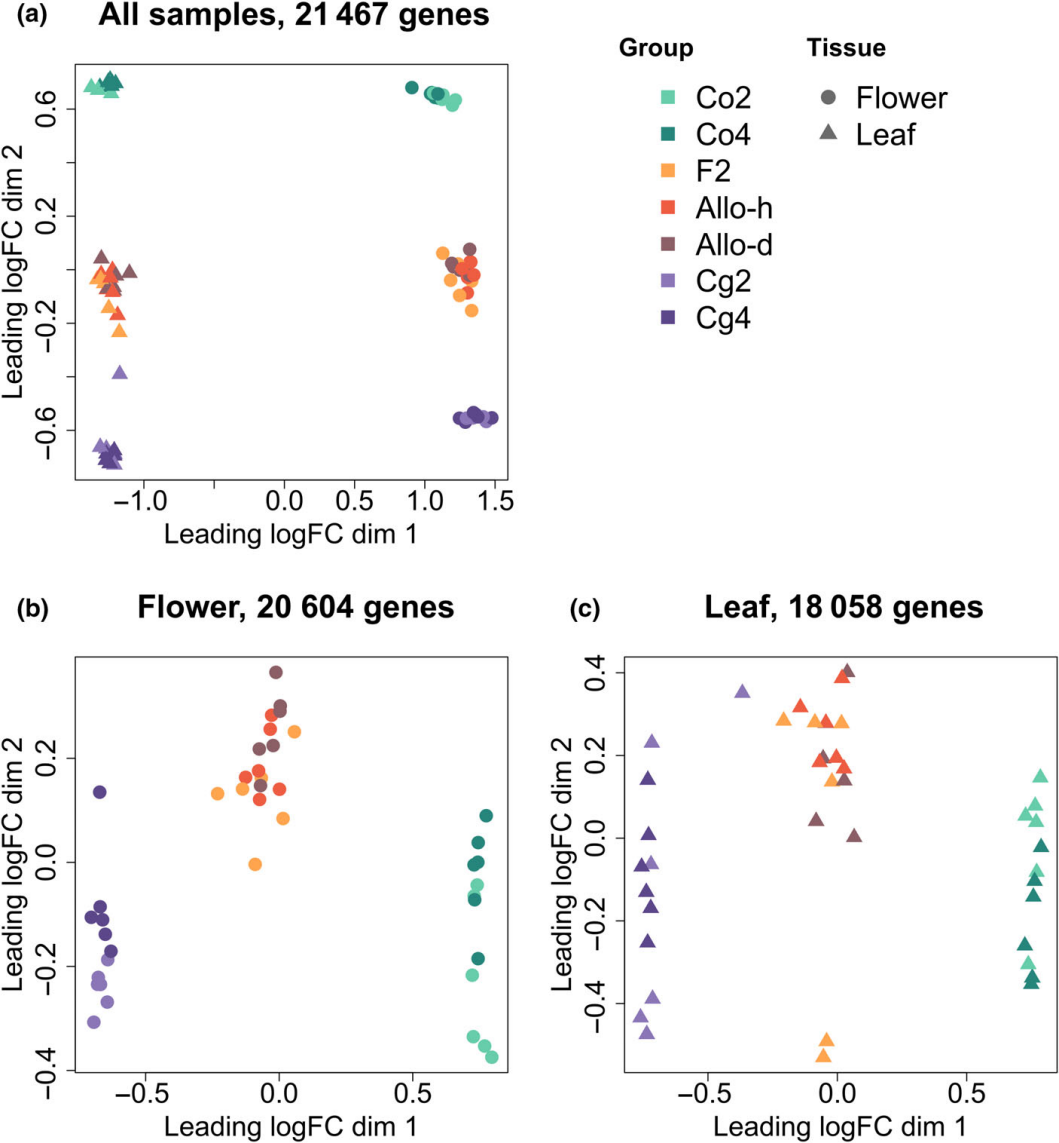
Transcriptome‐wide expression pattern visualized by multidimensional scaling (MDS) plots. The plots were made with (a) all samples, (b) only the flower samples, or (c) only the leaf samples. Genes with transcripts per million (TPM) > 2 in at least three samples were used for the analysis, and the expression levels were normalized with the trimmed mean of *M*‐values (TMM) method.

In total, 21 467 467 genes were expressed (TPM > 2) in at least three samples. The transcriptome‐wide gene expression pattern of all samples was visualized by an MDS plot (Fig. 3a). The samples were grouped by tissues in the first dimension. In the second dimension, the flower and leaf samples had symmetrical patterns, where the samples were grouped by species, and the diploid samples were mixed with the corresponding tetraploid samples.

Two separate MDS plots were further made with only the flower (Fig. 3b) or leaf samples (Fig. 3c). The samples were still grouped by species within each tissue, with (Co2 + Co4) and (Cg2 + Cg4) being the two most distinct clusters and hybrids being in the middle. The second dimension displayed variation within each of the three clusters, yet the diploid and tetraploid samples were still mixed in most clusters (Fig. 3a–c). Only in flowers were the Cg2 and Cg4 groups slightly differentiated along the second axis.

### Differential expression analysis

With a threshold of FC > 2 and FDR < 0.05, we identified hundreds or thousands of DEGs in most of the group pairs, except for the comparisons between diploids and the corresponding tetraploids or between different hybrid groups (Figs S4, S5). The analyses in leaves showed a similar pattern as in flowers, but fewer DEGs were found.

Differentially expressed genes were abundant in all comparisons between a hybrid group and a parental group (Figs S4, S5). The number of hybrid–parent DEGs ranged from 359 to 962 in flowers and 225 to 627 in leaves. The majority of these hybrid–parent DEGs was shared among the three hybrid groups, and, importantly, most of these DEGs were already differentially expressed between the two parental groups (Fig. S6), suggesting that the outcome of hybridization occurs rather deterministically. In all hybrid–parent contrasts, there were more upregulated DEGs than downregulated DEGs.

In contrast to the large number of DEGs observed in the hybrids–parent pairs, we found almost no DEGs between diploids and the corresponding autotetraploids (Figs S4, S5). All the diploid–tetraploid contrasts had no more than 11 DEGs in both tissues, showing the limited effect of WGD on expression profiles.

### Nonadditively expressed genes were highly shared among hybrid groups and with parental differentially expressed genes

Both TE‐mediated genomic shock and interactions of diverged regulatory elements are expected to cause nonadditive gene expression in diploid or tetraploid hybrids. In particular, if nonadditive gene expression is mainly caused by TE transpositions, we expect that genes showing nonadditive expression occur randomly among lines and are independent of expression divergence between the diploid parental species.

To test these expectations, we identified nonadditively expressed genes (complete ELD or TRE) in each of the three hybrid groups (F2, Allo‐d, and Allo‐h), using the results of the DE analyses (Table S5). On average, 6.3% and 4.4% of the genes in the three hybrid groups showed complete ELD in flowers and leaves, respectively. The percentage of ELD genes only slightly differed among the three hybrid groups (Flower: F2, 6.6%; Allo‐d, 6.4%; Allo‐h, 6.0%; Leaf: F2, 5.0%; Allo‐d, 4.2%; Allo‐h, 4.1%). Almost no TRE gene was found in any hybrid group (< 0.02% in all groups and both tissues).

**Fig. 4:**
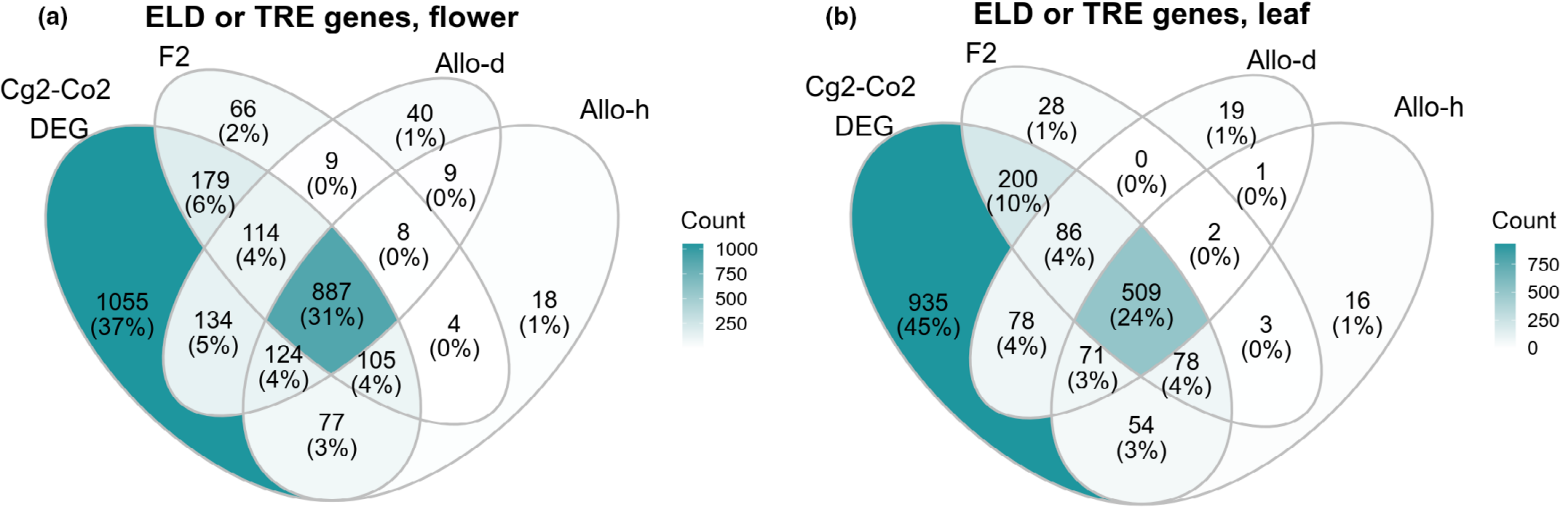
Venn diagram analyses of nonadditively expressed genes in the three hybrid groups (F2, Allo‐d, and Allo‐h), compared with parental expression‐differential (PED) genes (Cg2–Co2 DEG). Both nonadditively expressed genes and PED genes were identified by differential expression analyses (fold‐change > 2, false discovery rate (FDR) < 0.05). Genes showing complete expression level dominance (ELD) or transgressive expression (TRE) in each group were considered as nonadditive gene expression and were compared in flowers (a) or leaves (b).

Most nonadditively expressed genes were shared among the three hybrid groups and overlapped with PED genes (Fig. 4), again showing that nonadditive gene expression in neo‐hybrids is not stochastic and is unlikely to be caused by new TE transpositions.

### Synthetic diploid hybrids, autotetraploids, or allotetraploids did not show an excess of extreme gene expression

**Fig. 5:**
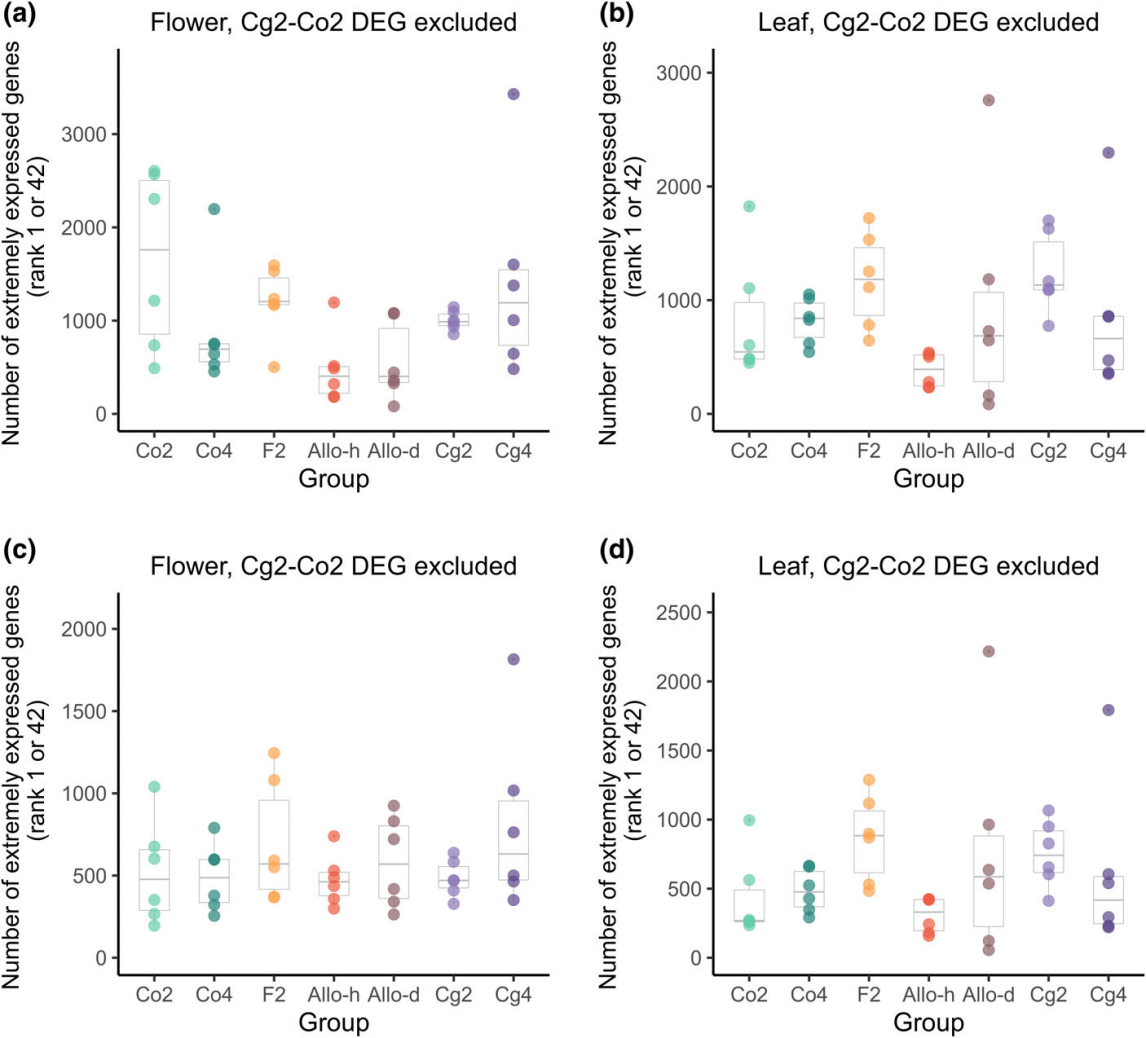
Extremely expressed genes per individual. The number of extremely expressed genes (rank 1 or 42) was counted for each of the 42 individuals from the seven groups, using all the genes with count‐per‐million (CPM) > 1 in at least two samples (a, b), or only the genes which were not differentially expressed between the Co2 and Cg2 groups (c, d). Each dot represents one individual. Within each box plot, the bold horizontal line represents the median value; box range means values within interquartile range (IQR) from first quartile (Q1) to third quartile (Q3); up and down whiskers indicate 1.5 IQR above the Q3 (Q3+1.5 IQR) and 1.5 IQR below the Q1 (Q1−1.5 IQR), separately; circles representative outliers.

To test whether there was more individual‐specific extreme gene expression after hybridization or WGD, the number of extremely expressed genes was compared among the seven groups at the individual level. The expression levels of each gene were compared among the 42 individuals of the seven groups, and rank 1 or 42 was regarded as extreme expressions. Only slight differences were found among the seven groups (Fig. 5a,b; one‐way ANOVA; Flower: *F*_6,35_ = 2.64, *P* = 0.032; Leaf: *F*_6,35_ = 1.52, *P* = 0.200). The synthetic diploid hybrids, autotetraploids, or allotetraploids did not show an excess of extremely expressed genes. The numbers of extremely expressed genes in these groups were similar to or lower than in the parental groups.

Since the additive expression in hybrids would result in midparent values, the potential dysregulation caused by TE‐reactivation or chromosomal rearrangement could be masked by a decrease in extreme expression due to additive expression in hybrids. To mitigate the influence of this midparent effect, we did the same analysis again with only the genes that were not differentially expressed between the Cg2 and Co2 groups. Again, the number of extremely expressed genes did not differ much among the seven groups (Fig. 5c,d; one‐way ANOVA; Flower: *F*_6,35_ = 1.05, *P* = 0.411; Leaf: *F*_6,35_ = 1.33, *P* = 0.270). The synthetic diploid hybrids, autotetraploids, or allotetraploids did not show a general elevation of extreme expression.

### No sign of large‐scale TE‐reactivation in synthetic diploid hybrids, autotetraploids, or allotetraploids

**Fig. 6:**
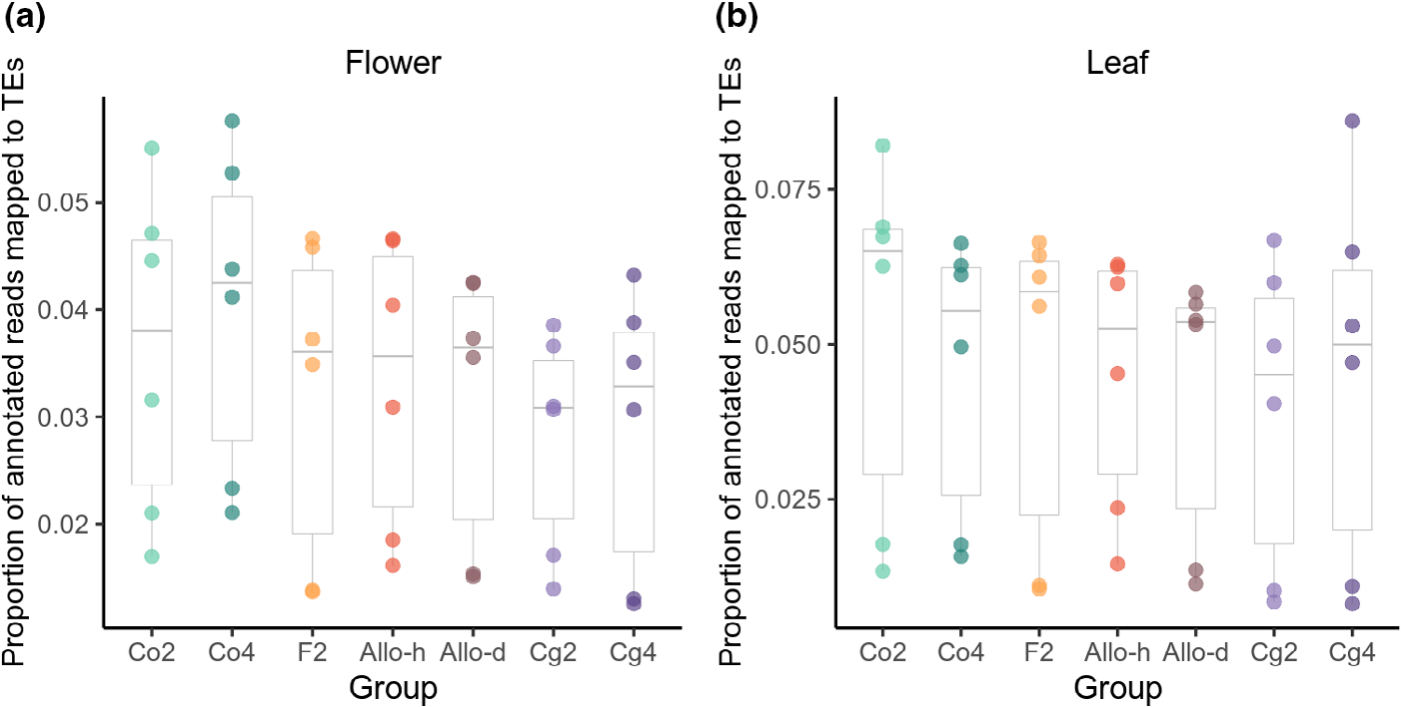
Abundance of transposable elements (TE) in transcriptomes. The proportion of annotated RNA‐sequencing reads that were mapped to TE sequences (reads mapped to TEs/reads mapped to genes or TEs) was calculated for each individual and compared among the seven plant groups in flowers (a) and leaves (b). Each dot represents one individual. Within each box plot, the bold horizontal line represents the median value; box range means values within interquartile range (IQR) from first quartile (Q1) to third quartile (Q3); up and down whiskers indicate 1.5 IQR above the Q3 (Q3+1.5IQR) and 1.5 IQR below the Q1 (Q1−1.5 IQR), separately; circles representative outliers.

Under a TE‐mediated genomic shock scenario, both transcriptional activation and new transposition of TEs are expected. To test whether there was a large‐scale TE transcriptional reactivation after hybridization or WGD, the total expression level of all TEs was compared between diploid parental species and synthetic hybrids and polyploids. Although we can only examine common TEs that can be annotated in the *C. rubella* reference genome, a large‐scale TE‐reactivation should also be reflected by these common TEs. In total, 42 509 partial or complete TE sequences were marked on the *C. rubella* reference genome, corresponding to 1020 different TE sequences. After downsampling, on average, 18.0 ± 0.3 million read pairs and 14.6 ± 0.3 million read pairs were annotated for each flower and leaf sample, respectively (Fig. S7). Among the annotated reads, 3.3% of flower reads and 4.5% of leaf reads were mapped to the TE sequences (Fig. 6).

Neither hybridization nor WGD significantly affected the proportion of TEs in the transcriptomes of either flowers or leaves (Table S6; Fig. 6). We obtained the same results by repeating separately the analyses on several TE categories (LTRs, Helitrons, SINEs, and LINEs; Fig. S8, GLM, quasi‐binomial, *P* > 0.1 for hybridization, polyploidization, and their interactions in all eight tests) suggesting that no category was activated by WGD or hybridization.

We found a batch/lane effect on the relative abundance of TE (Table S6). The samples sequenced on one of the three lanes had a much lower proportion of TEs transcripts than the other two lanes (lane TH‐2637: 5.3 ± 0.2%; TH‐2638: 1.5 ± 0.1%; and TH‐2639: 4.8 ± 0.2%). Nonetheless, since we used a balanced block design, that is, each plant group and tissue had two samples on each of the three lanes and included the batch/lane effect in all the GLM models, the batch/lane effect should not strongly bias the comparison among plant groups.

## Discussion

### The relative importance of polyploidization and hybridization in allopolyploidization

Relative gene expression pattern was shifted in all diploid or tetraploid hybrids (Figs 3, S4, S5). By contrast, the effect of WGD was strikingly small. Hundreds of DEGs were identified from each hybrid–parent comparison, but no more than 11 DEGs (0.056% genes) in the eight diploid–tetraploid comparisons. Hence, hybridization, rather than WGD, was the main source of relative expression changes in neo‐allopolyploids, in agreement with earlier predictions (Leitch & Leitch, 2008; Tayalé & Parisod, 2013). Similar results were obtained in *Arabidopsis*, cotton, and maize, either by comparing allopolyploids with homoploid hybrids (Chaudhary *et al*., 2009) or by comparing allopolyploids with autopolyploids (Wang *et al*., 2006; Riddle *et al*., 2010). In *Arabidopsis*, more than a 1000 genes are differentially expressed between the resynthesized allotetraploids and the midparent value of the autotetraploid parents, while only 88 genes are differentially expressed between the diploid and autotetraploid plants of one parental species (Wang *et al*., 2006). Our results are in line with these studies, but with a full factorial design, we were also able to separate the main effects of WGD and hybridization from their interactions, confirming that the shift of the expression pattern was mainly caused by hybridization *per se*.

WGD often has a limited immediate impact on gene expression patterns in synthetic autopolyploids, affecting the expression of a small fraction (< 1%) of genes (Galitski *et al*., 1999; Wang *et al*., 2006; Allario *et al*., 2011), or affecting a larger fraction of genes but with few expression changes larger than twofold (Stupar *et al*., 2007; Riddle *et al*., 2010; Yu *et al*., 2010; Yin *et al*., 2018). Moderate levels of expression changes were also found in some autopolyploids, where 2–5% of genes showed an FC larger than two (Wu *et al*., 2014; Dai *et al*., 2015; Xiang *et al*., 2019). It is worth noting that the relative importance of WGD may change over time. Studies that found WGD having minimal effects on gene expression were from newly generated autopolyploids (Galitski *et al*., 1999; Wang *et al*., 2006), whereas some studies on older autopolyploid species did find larger differentiation of gene expression patterns (e.g. Visger *et al*., 2019). Besides, in the newly generated *Capsella* autotetraploids, despite the trivial change in relative gene expression pattern, we noticed that WGD still had conspicuous effects on phenotypes (Fig. 2a–f), possibly through the change in physical attributes, such as cell size or absolute concentrations for vegetative and developmental traits (Visger *et al*., 2019; Xu *et al*., 2020), or chromosome behaviors during meiosis for pollen viability. Considering these two observations, the change in expression pattern found in older autopolyploids could be the result of long‐term evolution induced by the polyploid state, rather than instantly triggered by WGD. In this sense, it is still possible for WGD to play a larger role in allopolyploids in the long term.

Our study has two small caveats: first, we only used one line from each diploid parental species to generate all polyploids and hybrids, although the transcriptomic reaction to WGD can be genotype‐specific (Yu *et al*., 2010). Second, we did not perform reciprocal crosses but only used *C. orientalis* as the maternal plants to mimic the natural allotetraploid *C. bursa‐pastoris*, however the impact of hybridization can depend on cross direction (Lafon‐Placette *et al*., 2018). Nevertheless, considering the magnitude of the variation caused by genotype or cross direction, it is likely to be the general case that the initial expression pattern of allopolyploids is determined by interspecific hybridization, rather than WGD.

### No sign of TE‐mediated genomic shock after hybridization or WGD

We tested two expectations of the TE‐mediated genome shock scenario: a global TE‐reactivation and a large‐scale gene expression change due to transcriptional reactivation or new transposition of TEs. In *Capsella*, neither hybridization nor WGD significantly increased TE transcription (Fig. 6; Table S6). Almost no gene was differentially expressed between diploids and the corresponding autotetraploids (Figs S4, S5). Hence, WGD alone is unlikely to cause a TE‐mediated genome shock in *Capsella*. What about hybridization? Up to 4–6% of genes showed clear nonadditive expression in diploid hybrids or allotetraploids. However, these gene expression changes were highly deterministic: the nonadditively expressed genes were largely shared among independent lines and among the three hybrid groups (F2, Allo‐d, and Allo‐h), and, importantly, overlapped with the DEGs between the two parental species (Fig. 4). In addition, individual‐specific gene dysregulation was not increased in hybrids either (Fig. 5). The deterministic outcome of hybridization indicates that TE transposition is not the main cause of nonadditive expression in hybrids, because transposition and transposition‐induced chromosomal restructuring are expected to have stochastic effects in independent lines. Although a global transcriptional reactivation of preexisting silenced TEs may also result in deterministic gene expression change, considering that we did not find evidence of elevated TE transcription in hybrids, a global transcriptional reactivation is less likely than another simpler explanation: nonadditive expression in hybrids is mainly caused by intergenomic interactions between divergent regulatory elements. Taken together, TE‐mediated genomic shock is unlikely to be a prominent mechanism in shaping the expression pattern in *Capsella* hybrids.

It is worth noting that we do not reject any contribution of TEs, but only an instant, stress‐induced global TE‐reactivation. TEs can still serve as regulatory elements (Feschotte, 2008; Chuong *et al*., 2016) and contribute to ‘intergenomic interactions between regulatory elements’ in hybrids. Results of previous studies on TE activity in diploid or polyploid hybrids vary. In some studies, TE activities or TE‐related structural variation were increased in hybrids, albeit to moderate levels, or were restricted to specific TE families (Parisod *et al*., 2009; Vicient & Casacuberta, 2017). No change in TE activity was observed in other studies (Ågren *et al*., 2016; Göbel *et al*., 2018; Cheng *et al*., 2019). Hence, altogether, many studies on hybrids and polyploids deviate from the original ‘genomic shock’ scenario (McClintock, 1984; Baduel *et al*., 2018). Instead of a stress‐induced global ‘shock’, the moderate increase in TE activity in some hybrids may be caused by the mismatch of unbalanced/divergent TE and TE‐suppressors in hybrids (Parisod *et al*., 2010) and therefore depends on both TE content and TE divergence in parental genomes. *Capsella* species and several other species in Brassicaceae have relatively lower TE content than some model species in Poaceae (Kaul *et al*., 2000; Schnable *et al*., 2009; Slotte *et al*., 2013; Mirouze & Vitte, 2014). This difference in TE content mirrors the contrast between the minimum TE effects on hybrids in *Capsella* or *Arabidopsis* (Beaulieu *et al*., 2009) and more obvious TE activity in maize (Estep *et al*., 2013) or rice (Liu & Wendel, 2000).

In the natural allotetraploid species, *C. bursa‐pastoris*, the genome‐wide TE content is not higher than that of the two parental species (Douglas *et al*., 2015; Ågren *et al*., 2016), although, in gene‐rich regions, retrotransposons were more abundant in *C. bursa‐pastoris* than in both parents (Ågren *et al*., 2016). Our results showed that a large‐scale TE‐reactivation is unlikely, even at the birth of this allotetraploid species, supporting that *C. bursa‐pastoris* did not experience a TE‐mediated genomic shock, rather than recovered from a shock by later TE elimination. The elevated retrotransposon abundance in gene‐rich regions of natural *C. bursa‐pastoris* is more likely the result of a gradual accumulation of TE under relaxed purifying selection, as suggested by previous studies (Ågren *et al*., 2016; Baduel *et al*., 2019).

Finally, we would like to stress that previous mixed conclusions on genomic shock partly come from the broad use of the term ‘genomic shock’ to refer to distinct phenomena, mechanisms, or timescales. Communication would be facilitated by the use of more specific terms instead of gathering diverse hypotheses under the umbrella of ‘genomic shock’.

### Interactions between WGD and hybridization

The coupling of WGD and hybridization is so common in nature that it is easy to forget that they are distinct processes with potential interactions. Here, we aimed to explore whether the high frequency of allopolyploid species could be alternatively explained by short‐term interactions between WGD and hybridization, reducing the negative effects of the two processes.

The interaction between WGD and hybridization was significant for pollen viability (Fig. 2d; Table S3). Both hybridization and WGD alone decreased pollen viability, but WGD was less deleterious for hybrids. Pollen viability is a good indicator of meiotic abnormalities (Srivastava *et al*., 1992; Jiang *et al*., 2013); hence, the interaction found in pollen viability is most likely caused by meiotic mechanisms. For diploid hybrids, WGD can provide homologous chromosomes for better meiotic pairing. In accordance with this expectation, pollen viability of the ‘hybridization‐first’ allotetraploids was slightly higher than for diploid hybrids. In the other direction, theoretically, hybridization may also improve the meiosis behavior of autopolyploids, since possessing differentiated homeologous chromosomes can reduce the rate of multivalents or promote preferential pairing (Ramsey & Schemske, 2002). We found that pollen viability of ‘hybridization‐first’ allotetraploids was higher than for autotetraploid *C. orientalis*, but still lower than in autotetraploid *C. grandiflora*. One possible explanation is that the self‐fertilizing *C. orientalis* has higher homozygosity than the outcrossing *C. grandiflora* (Douglas *et al*., 2015). The higher affinity among the four sets of chromosomes may lead to more severe meiotic problems in the autotetraploid *C. orientalis*, which will then benefit more from hybridization. This remains to be tested.

A similar interaction between WGD and hybridization was revealed in the proportion of normal seeds, but the beneficial effect of WGD is stronger in seeds (Fig. 2f; Table S3) than in pollen. With WGD, the proportion of normal seeds in hybrids was increased from 0.57 ± 0.03 (F2 group) to 0.78 ± 0.02 (Allo‐h group). The difference between pollen and seeds suggests that components other than meiotic mechanisms may contribute to the interaction. One possible contributor is the stoichiometric balance maintained by allopolyploidization. The parental dosage imbalance in endosperm is the major contributor to the hybrid barrier between *C. grandiflora* and *C. orientalis* (Lafon‐Placette *et al*., 2018). This endosperm defect would persist in the following generations of diploid hybrids, as the independent assortment of homeologous chromosomes will keep generating gametes with various hybridity. By contrast, for polyploid hybrids, WGD would inhibit independent assortment in hybrids and reduce the dosage variation among gametes, thus alleviating the dosage imbalance in endosperms.

Improving meiotic paring and maintaining dosage balance are two candidate mechanisms for the beneficial interaction between WGD and hybridization, both of which are short‐term organism‐level mechanisms. The success of an allopolyploid population also depends on long‐term mechanisms, such as higher genetic diversity, temporarily relaxed purifying selection, cytotype exclusion, and the establishment of reproductive isolation. Compared with those long‐term mechanisms, the relative importance of the identified instant interactions for the future of a newly created polyploid remains an open question.

### The pathway toward allopolyploidy matters

The prevalence or the influence of the order of WGD and hybridization in natural allopolyploid species has not been formally assessed. We found that the way in which allopolyploids are generated affects their performance. In particular, the ‘hybridization‐first’ and ‘doubling‐first’ *Capsella* allotetraploids differed in flowering time and pollen viability. Essentially, the key difference is that our polyploidization‐first allotetraploids had spent one extra generation as autotetraploids. The chromosomal rearrangements caused by meiosis in autopolyploids may be retained in the ‘doubling‐first’ allotetraploids and have a lasting influence. This phenomenon needs to be validated by examining the structural variation of the two forms of allopolyploids. A practical implication of this observation is that the way of resynthesizing allopolyploids may bias experimental results and testing both ways is desirable.

### Conclusions

This study takes advantage of a full factorial design provided by a series of synthetic *Capsella* hybrids and polyploids to examine the short‐term effects of WGD and hybridization on gene expression and phenotype. We showed that the initial gene expression pattern of allopolyploids was mainly determined by hybridization. While WGD had only a trivial effect on relative gene expression patterns, it still significantly affected phenotypes. Neither WGD nor hybridization was likely to trigger a TE‐mediated genomic shock in *Capsella*. In addition, WGD and hybridization had beneficial interactions on pollen and seed quality. By dissecting the confounding effects of WGD and hybridization, our study provides a solid basis for understanding the early stage of the evolution of allopolyploids.

## Author contributions

TD, AS, SG and ML designed the study. TD and AS performed experiments. TD analyzed the data. TD drafted the manuscript and SG, AS and ML edited it. All authors read and approved the final version of the manuscript.

## Supporting information


**Fig. S1** Examined *Capsella* pollen grains and seeds.
**Fig. S2** Number of mapped and annotated RNA‐sequencing read pairs before and after downsampling.
**Fig. S3** Transcriptome‐wide gene expression pattern visualized by principal component analysis.
**Fig. S4** Differentially expressed genes in pair‐wise contrasts of the seven plant groups in flower.
**Fig. S5** Differentially expressed genes in pair‐wise contrasts of the seven plant groups in leaf.
**Fig. S6** Venn diagram analyses of differentially expressed genes.
**Fig. S7** Number of RNA‐sequencing read pairs counted by software TEcount, before and after downsampling.
**Fig. S8** Expression level of major categories of transposable elements in the seven plant groups.
**Methods S1** Ovule rescue.
**Methods S2** Plant growing conditions.
**Methods S3** Examining pollen viability.
**Methods S4** RNA extraction.
**Methods S5** Downsampling mapped read pairs.
**Table S1** Primers of the PCR marker for verifying interspecific hybrids.
**Table S2** Classification of additive and nonadditive gene expression patterns in diploid hybrids and allotetraploids.
**Table S3** Effects of whole‐genome duplication and hybridization on pollen viability and the proportion of normal seeds.
**Table S5** Additive and nonadditive gene expression in diploid hybrids and allotetraploids.
**Table S6** Effects of whole‐genome duplication and hybridization on the proportion of transposable elements in flower and leaf transcriptomes.

**Table S4** RNA‐sequencing information.
Please note: Wiley is not responsible for the content or functionality of any Supporting Information supplied by the authors. Any queries (other than missing material) should be directed to the *New Phytologist* Central Office.

## Data Availability

All RNASEQ data are stored on SRA BioProject PRJNA848625. The biosample IDs are included in Table S4.
