# Supplementary material for: Expression pattern of resynthesized allotetraploid Capsella is determined by hybridization, not whole‐genome duplication
Source: New Phytol. 2022 Nov 7;237(1):339–53. doi: 10.1111/nph.18542 (PMC10099941; doi:10.1111/nph.18542)
Supplement: Supplementary file 1 — Fig. S1 Examined Capsella pollen grains and seeds. Fig. S2 Number of mapped and annotated RNA‐sequencing read pairs before and after downsampling. Fig. S3 Transcriptome‐wide gene expression pattern visualized by principal component analysis. Fig. S4 Differentially expressed genes in pair‐wise contrasts of the seven plant groups in flower. Fig. S5 Differentially expressed genes in pair‐wise contrasts of the seven plant groups in leaf. Fig. S6 Venn diagram analyses of differentially expressed genes. Fig. S7 Number of RNA‐sequencing read pairs counted by software TEcount, before and after downsampling. Fig. S8 Expression level of major categories of transposable elements in the seven plant groups. Methods S1 Ovule rescue. Methods S2 Plant growing conditions. Methods S3 Examining pollen viability. Methods S4 RNA extraction. Methods S5 Downsampling mapped read pairs. Table S1 Primers of the PCR marker for verifying interspecific hybrids. Table S2 Classification of additive and nonadditive gene expression patterns in diploid hybrids and allotetraploids. Table S3 Effects of whole‐genome duplication and hybridization on pollen viability and the proportion of normal seeds. Table S5 Additive and nonadditive gene expression in diploid hybrids and allotetraploids. Table S6 Effects of whole‐genome duplication and hybridization on the proportion of transposable elements in flower and leaf transcriptomes. [file NPH-237-339-s002.pdf]

New Phytologist

***New Phytologist* Supporting Information**

Article title: Expression pattern of resynthesized allotetraploid *Capsella* is determined by hybridization, not whole genome duplication
Authors: Tianlin Duan, Adrien Sicard, Sylvain Glémin, Martin Lascoux
Article acceptance date: 04 October 2022

The following Supporting Information is available for this article:

Fig. S1 Examined *Capsella* pollen grains and seeds.

Fig. S2 Number of mapped and annotated RNA-sequencing read pairs before and after downsampling.

Fig. S3 Transcriptome-wide gene expression pattern visualized by principal component analysis (PCA)

Fig. S4 Differentially expressed genes in pair-wise contrasts of the seven plant groups in flower

Fig. S5 Differentially expressed genes in pair-wise contrasts of the seven plant groups in leaf.

Fig. S6 Venn diagram analyses of differentially expressed (DE) genes.

Fig. S7 Number of RNA-sequencing read pairs counted by software TEcount, before and after downsampling.

Fig. S8 Expression level of major categories of transposable elements in the seven plant groups.

Table S1 Primers of the PCR marker for verifying interspecific hybrids

Table S2 Classification of additive and non-additive gene expression patterns in diploid hybrids and allotetraploids

Table S3 Effects of whole-genome duplication (WGD) and hybridization (HYB) on pollen viability and the proportion of normal seeds.

Table S4 RNA-sequencing information (see the separate file)

Table S5 Additive and non-additive gene expression in diploid hybrids and allotetraploids

Table S6 Effects of whole genome duplication (WGD) and hybridization (HYB) on the proportion of transposable elements (TE) in flower and leaf transcriptomes

Methods S1 Ovule rescue

Methods S2 Plant growing conditions

Methods S3 Examining pollen viability

Methods S4 RNA extraction

Methods S5 Downsampling mapped read pairs

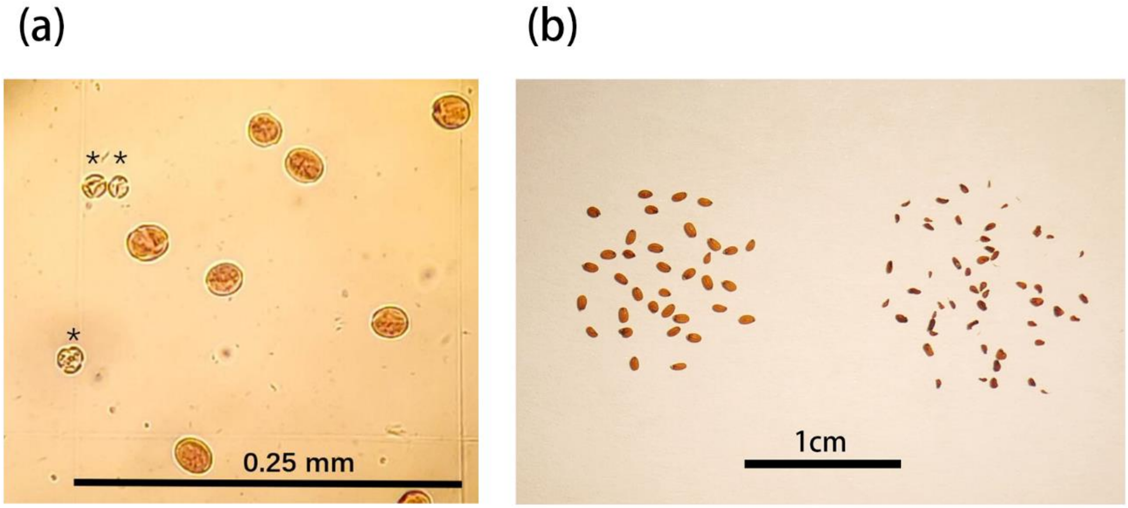

Fig. S1 Examined *Capsella* pollen grains and seeds. (a) Pollen grains stained with aceto-carmine solution. Pollen grains staining pink were classified as viable, and the transparent or the greenish-yellow ones were considered non-viable (marked with stars). (b) Seeds that were flat or small and dark were regarded as “abnormal seeds” (right side), otherwise, the seeds were regarded as “normal seeds” (left side). The seeds on both sides were from the same individual (Allo-d-8-6).

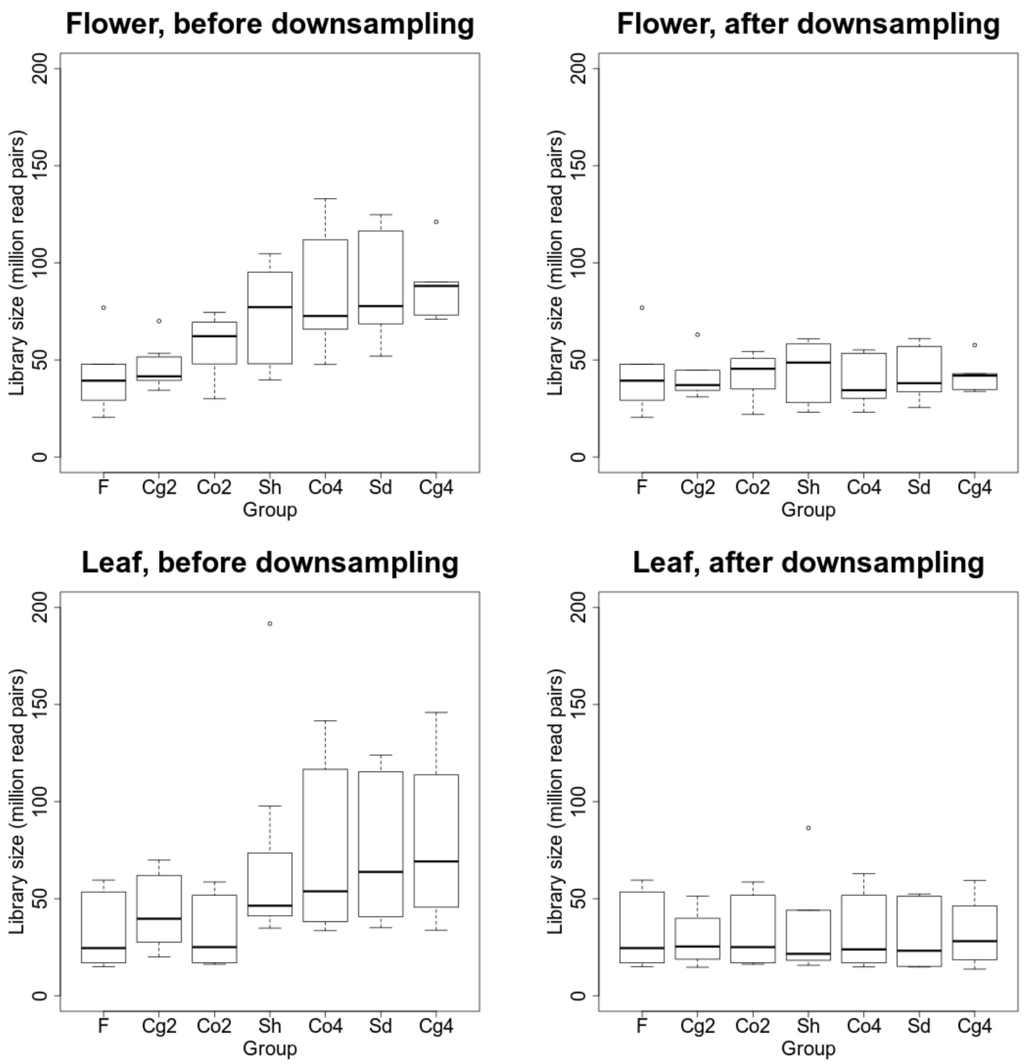

Fig. S2 Number of mapped and annotated RNA-sequencing read pairs before and after downsampling. The downsampled dataset was used for all the analyses on RNA-sequencing data except the analyses of transposable elements.

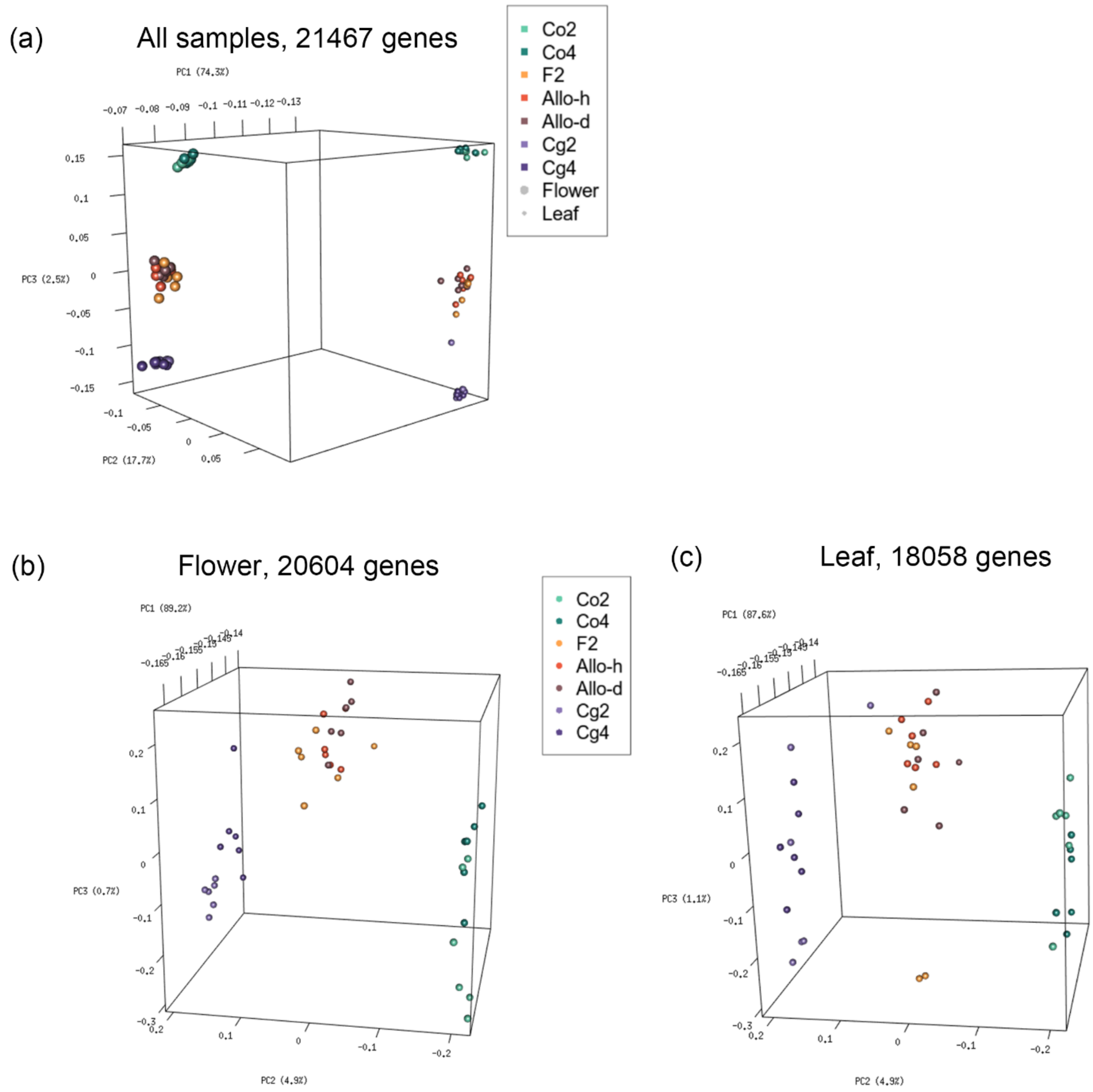

Fig. S3 Transcriptome-wide gene expression pattern visualized by principal component analysis (PCA). Principal analyses were performed with genes that had transcripts-per-million > 2 in at least three samples, using (a) all samples, (b) only flower samples, or (c) only leaf samples. Log-transformed TMM normalized TPM values were used for the analysis

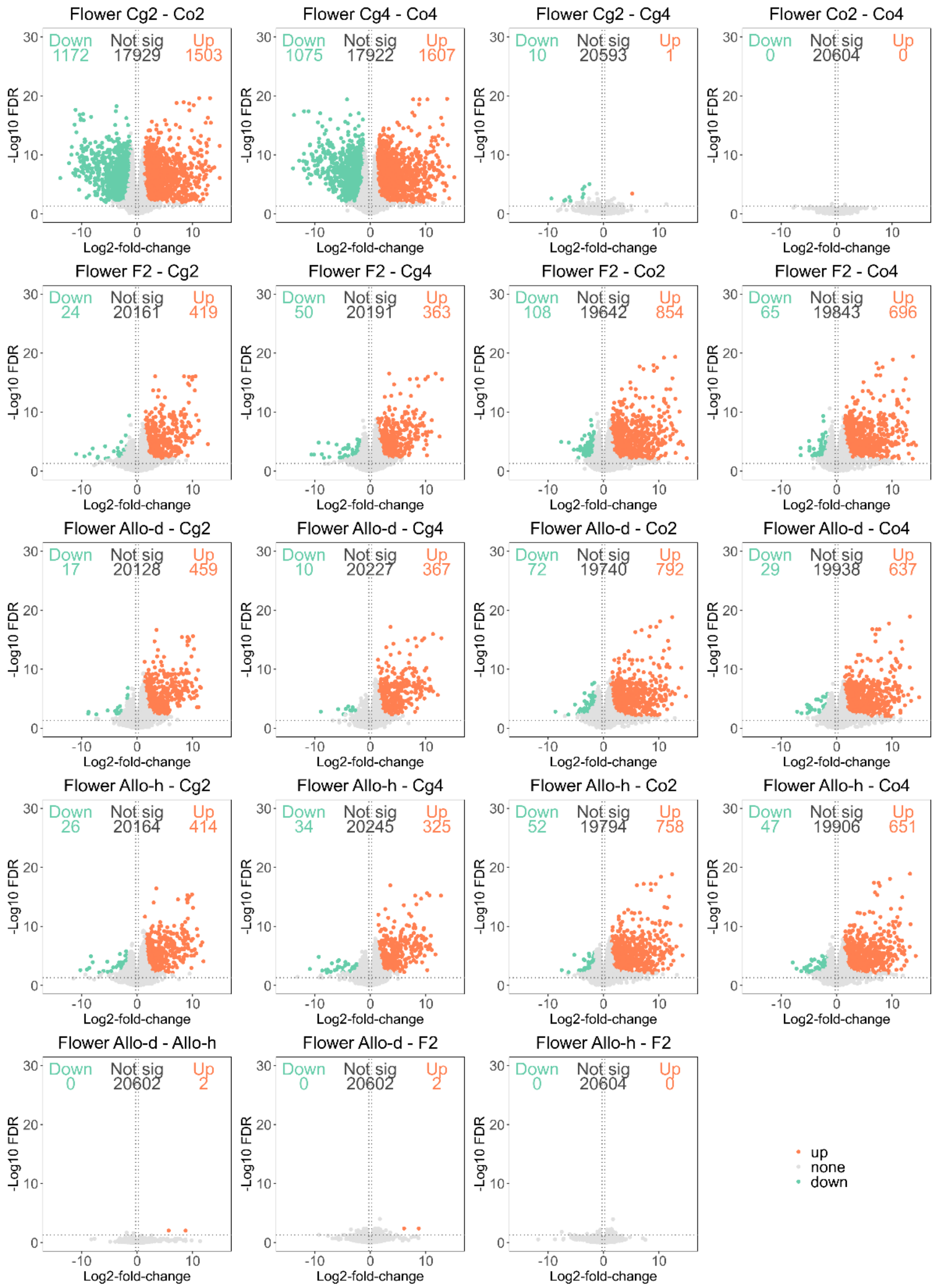

Fig. S4 Differentially expressed (DE) genes in pair-wise contrasts of the seven groups in flower. Genes with counts-per-million > 1 in at least two samples were used in the analysis, and the expression levels were normalized with the trimmed mean of M-values (TMM) method. DE genes with a fold-change > 2 and a false discovery rate (FDR) < 0.05 were considered significant.

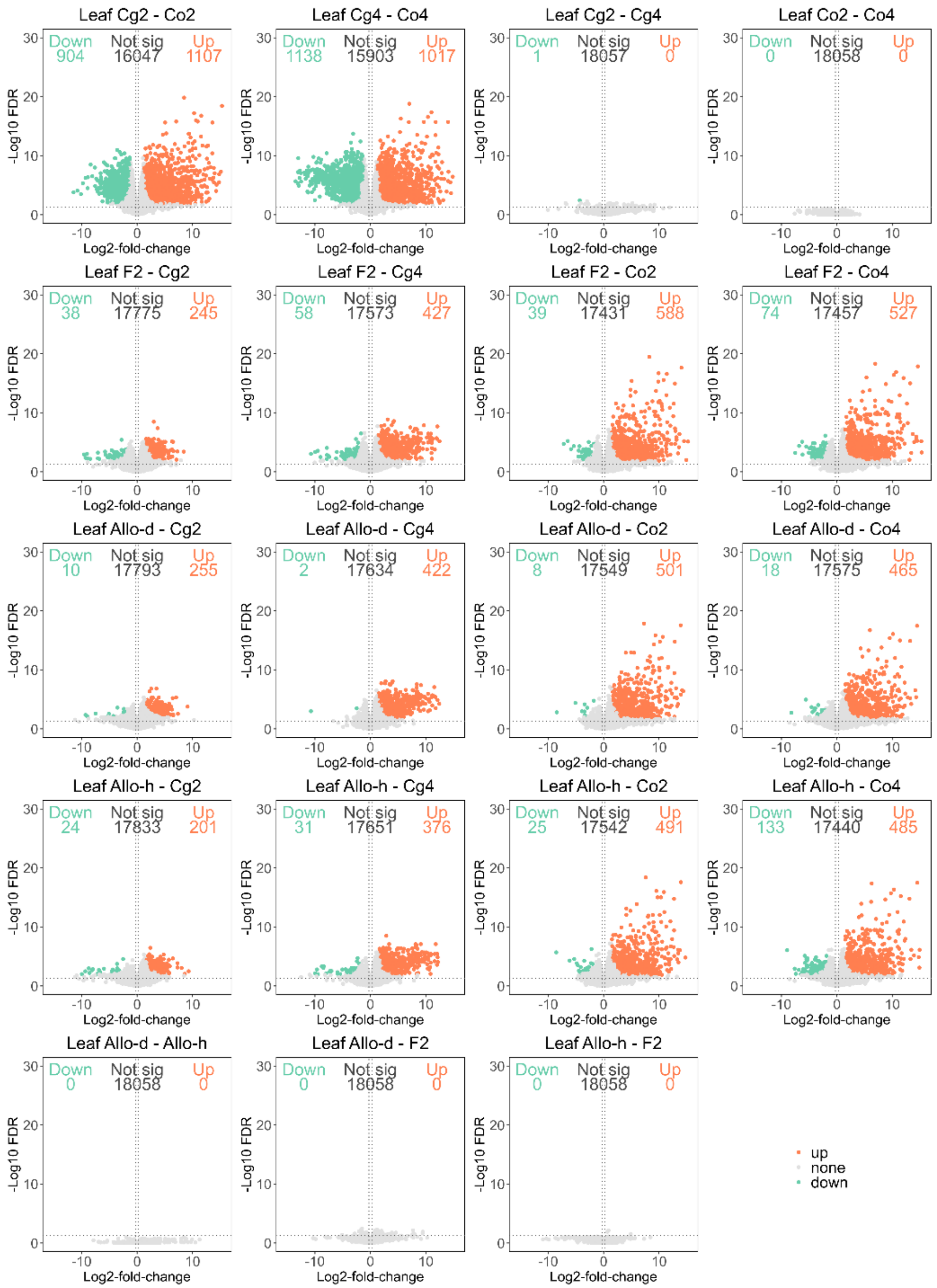

Fig. S5 Differentially expressed (DE) genes in pair-wise contrasts of the seven groups in leaf. Genes with counts-per-million > 1 in at least two samples were used in the analysis, and the

expression levels were normalized with the trimmed mean of M-values (TMM) method. DE genes with a fold-change > 2 and a false discovery rate (FDR) < 0.05 were considered significant.

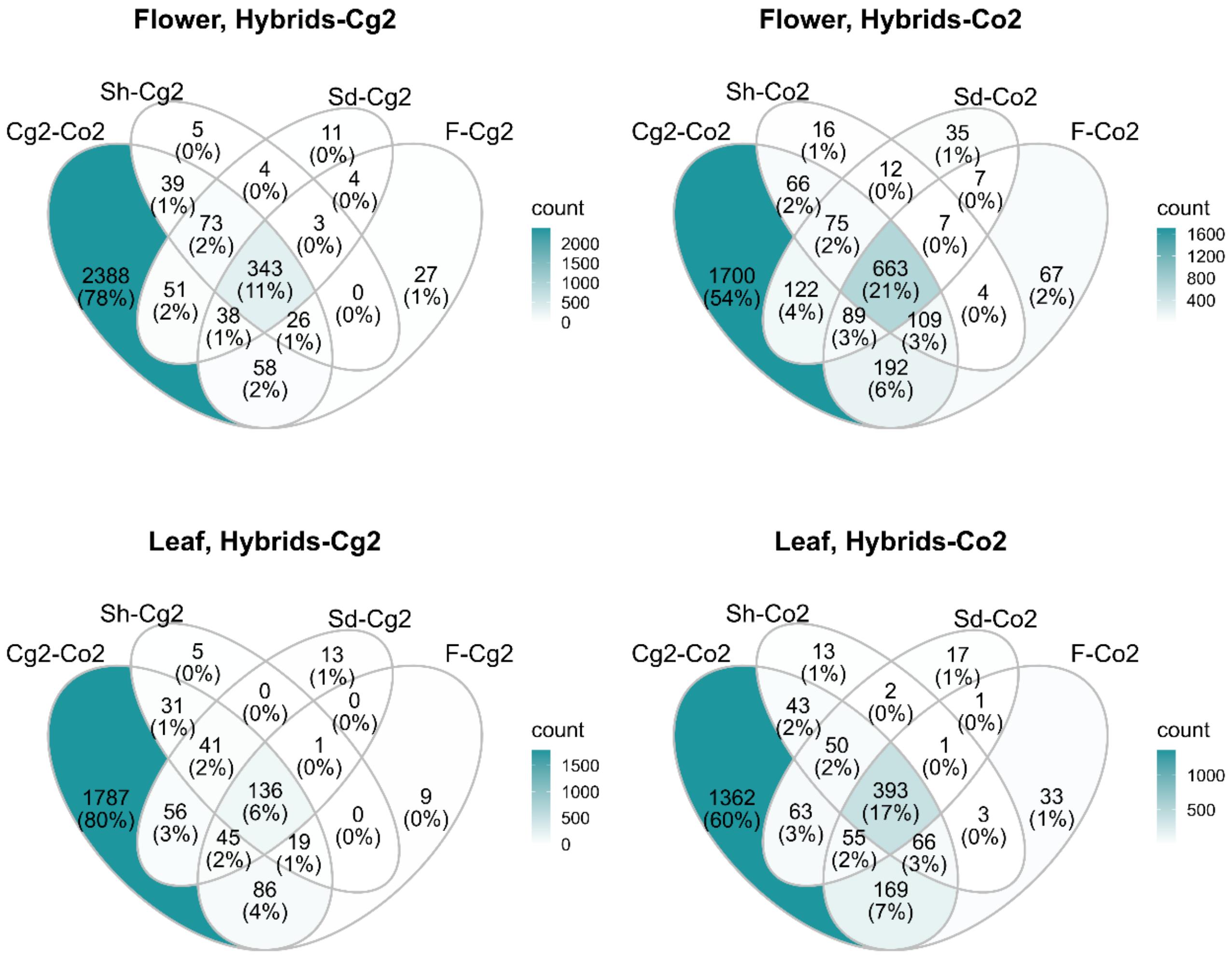

Fig. S6 Venn diagram analyses of differentially expressed (DE) genes. The hybrids-parent DE genes (fold-change > 2, false discovery rate (FDR) < 0.05) were compared among different hybrid groups and were also compared with the DE genes between the diploid parental groups.

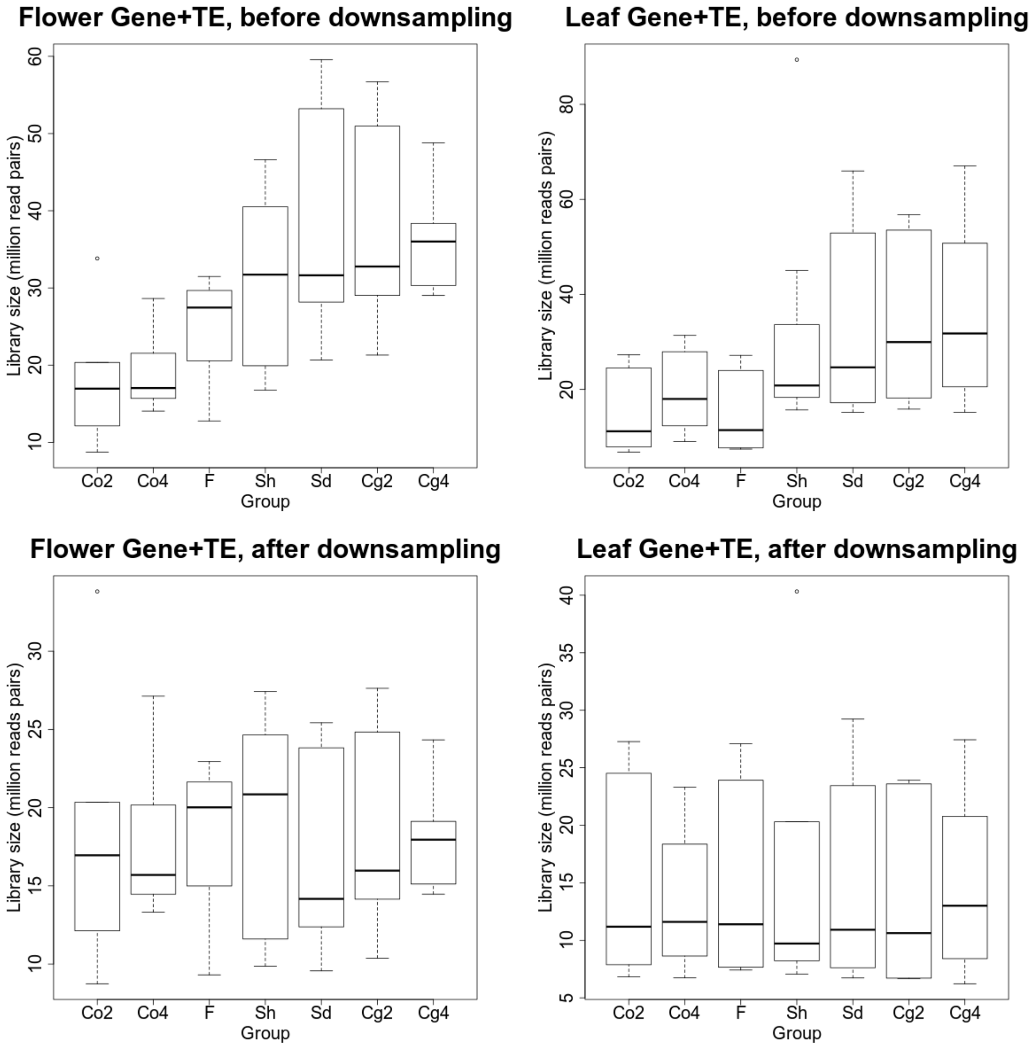

Fig. S7 Number of RNA-sequencing read pairs counted by software TEcount, before and after downsampling. This dataset was only used for analyses on transposable elements.

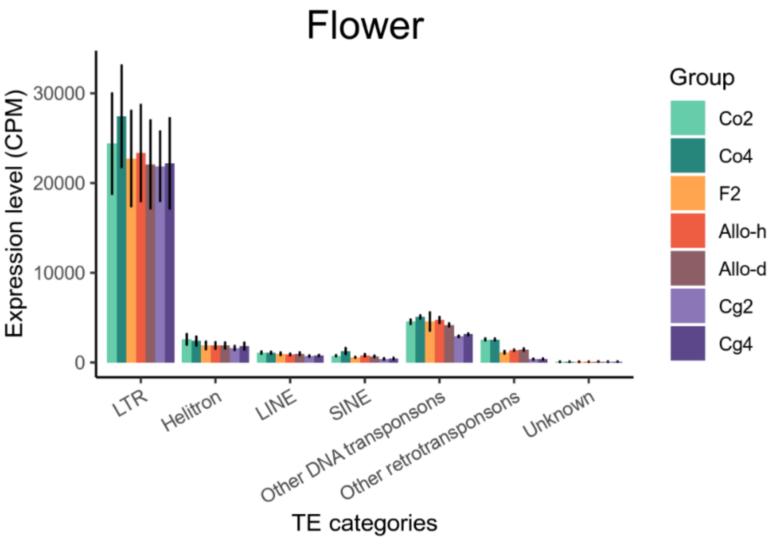

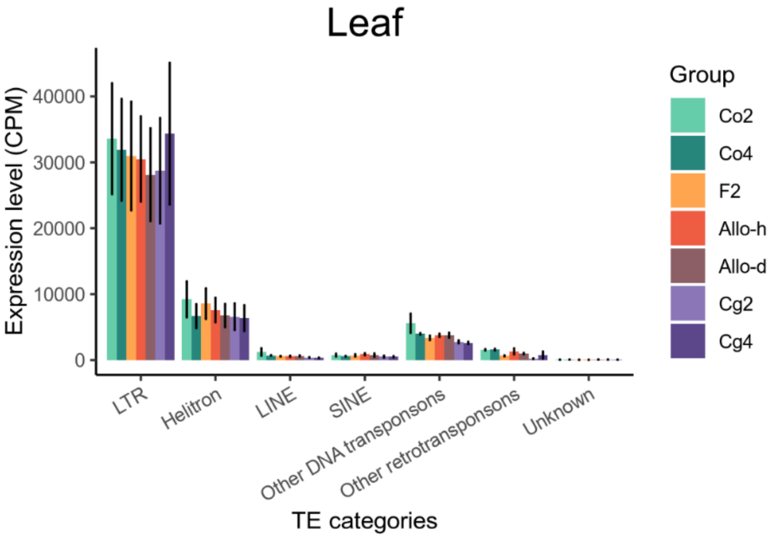

Fig. S8 Expression level of major categories of transposable elements in the seven plant groups. Expression levels were normalized by read counts per million (CPM). Error bars are the group mean ± standard error. The expression level of long terminal repeat (LTR), helitron, short interspersed nuclear elements (SINE) and long interspersed nuclear elements (LINE) were compared among the seven plant groups. In none of the four TE categories did hybridization or polyploidization have a significant effect on TE abundance (GLM, quasi-binomial, p-value > 0.1 for hybridization, polyploidization and their interactions in all eight tests).

Table S1 Primers of the PCR marker for verifying interspecific hybrids. It reveals a 103-bp indel that is fixed between C. orientalis and C. grandiflora.

Primer name	Sequence (5'->3')	Template strand
coac1-indel-F1	AGGCAACAACAAGCGTTACAA	Plus
coac1-indel-R1	GTGGAATCCGCAACCATGAC	Minus

Table S2 Classification of additive and non-additive gene expression patterns in diploid hybrids and allotetraploids

Group	Description	Classification criteria
a	Additive expression with no parental differentiation	$Cg_i = x_{ij} = Co_i$
b	Partial ELD or<br>additive expression with parental differentiation	($Cg_i < x_{ij} < Co_i$) or<br>($Co_i < x_{ij} < Cg_i$) or<br>($Cg_i \neq Co_i$ and $x_{ij} = Cg_i$ and $x_{ij} = Co_i$)
c	Up-regulated ELD toward Cg2	$x_{ij} = Cg_i$ and $x_{ij} > Co_i$
d	Down-regulated ELD toward Cg2	$x_{ij} = Cg_i$ and $x_{ij} < Co_i$
e	Up-regulated ELD toward Co2	$x_{ij} = Co_i$ and $x_{ij} > Cg_i$
f	Down-regulated ELD toward Co2	$x_{ij} = Co_i$ and $x_{ij} < Cg_i$
g	Up-regulated TRE with no parental differentiation	$Cg_i = Co_i$ and $x_{ij} > Cg_i$ and $x_{ij} > Co_i$
h	Up-regulated TRE with parental differentiation	$Cg_i \neq Co_i$ and $x_{ij} > Cg_i$ and $x_{ij} > Co_i$
i	Down-regulated TRE with no parental differentiation	$Cg_i = Co_i$ and $x_{ij} < Cg_i$ and $x_{ij} < Co_i$
j	Down-regulated TRE with parental differentiation	$Cg_i \neq Co_i$ and $x_{ij} < Cg_i$ and $x_{ij} < Co_i$

* Cgi: expression level of gene i in the Cg2 group; Coi: expression level of gene i in the Co2 group; xij: expression level of gene i in allotetraploid group j, and j∈(F2, Alllo-d, Allo-h); ELD: expression level dominance; TRE: transgressive expression; The significance of differential expression between groups were determined by the results of differential expression analysis, with a threshold of fold-change > 2 and false discovery rate < 0.05.

Table S3 Effects of whole-genome duplication (WGD) and hybridization (HYB) on pollen viability and the proportion of normal seeds.*

	**GLM**					
	**Flower**			**Leaf**		
	**df**	**F**	**p-value**	**df**	**F**	**p-value**
WGD	1	0.112	0.740	1	0.252	0.619
HYB	1	0.0667	0.798	1	0.120	0.731
WGD× HYB	1	0.113	0.738	1	0.193	0.663
Batch/lane effect	2	125	**<0.001**	2	176	**<0.001**
Residuals	36	-	-	36	-	-

*Pollen viability (number of viable and non-viable pollens) and the proportion of normal seeds in ten fruits (number of normal and abnormal seeds) were analyzed by generalized linear models (GLMs) with a quasi-binomial error distribution and a logit link function. WGD (diploid/tetraploid) and hybridization (hybrid/non-hybrid) were added as categorical explanatory variables. After identifying a significant interaction, the effect of WGD on pollen viability was further analyzed among hybrid and non-hybrid plants, respectively. In all models, the effects of variables were tested with F-tests. Significant effects (p-value < 0.05) were highlighted in bold. A dash ("-") indicates that the field is not applicable.

Table S5 Additive and non-additive gene expression in diploid hybrids and allotetraploids *

		Additive Expression*		Nonadditive expression							
				Expression-level dominance (ELD)				Transgressive expression (TRE)			
				Cg-ELD		Co-ELD		Over-TRE		Under-TRE	
	Expression category / Plant group	a	b	c	d	e	f	g	h	i	j
Flower	F2	17842 (86.6%)	1390 (6.7%)	849 (4.1%)	95 (0.5%)	403 (2.0%)	22 (0.1%)	3 (0.0%)	0	0	0
	Allo-d	17863 (86.7%)	1416 (6.9%)	787 (3.8%)	68 (0.3%)	452 (2.2%)	15 (0.1%)	3 (0.0%)	0	0	0
	Allo-h	17890 (86.8%)	1482 (7.2%)	754 (3.7%)	47 (0.2%)	409 (2.0%)	22 (0.1%)	0	0	0	0
Leaf	F2	16014 (88.7%)	1138 (6.3%)	586 (3.2%)	39 (0.2%)	245 (1.4%)	36 (0.2%)	0	0	0	0
	Allo-d	16025 (88.7%)	1267 (7.0%)	497 (2.8%)	7 (0.0%)	252 (1.4%)	8 (0.0%)	2 (0.0%)	0	0	0
	Allo-h	16025 (88.7%)	1299 (7.2%)	487 (2.7%)	25 (0.1%)	200 (1.1%)	21 (0.1%)	1 (0.0%)	0	0	0

* The ten gene expression categories were: a) additive expression with no parental differentiation, b) partial ELD or additive expression with parental differentiation, c) Up-regulated ELD toward diploid *C. grandiflora* (Cg2), d) Down-regulated ELD toward Cg2, e) Up-regulated ELD toward diploid *C. orientalis* (Cg2), f) Down-regulated ELD toward Co2, g) Up-regulated TRE with no parental differentiation, h) Up-regulated TRE with parental differentiation, i) Down-regulated TRE with no parental differentiation, g) Down-regulated TRE with parental differentiation. Partial expression-level dominance (expression level in allotetraploids was not the same as the mid-parent value but still in the middle of the two diploid groups) was included in category b.

Table S6 Effects of whole genome duplication (WGD) and hybridization (HYB) on the proportion of transposable elements (TE) in flower and leaf transcriptomes. *

		GLM								
		All plants			Hybrid plants			Non-hybrid plants		
		df	F	p-value	df	F	p-value	df	F	p-value
Pollen viability	WGD	1	1.20	0.275	1	0.795	0.375	1	49.2	**<0.001**
	HYB	1	45.8	**<0.001**	-	-	-	-	-	-
	WGD×HYB	1	18.9	**<0.001**	-	-	-	-	-	-
	Residuals	211	-	-	95	-	-	116	-	-
Proportion of normal seeds	WGD	1	8.02	**5.14e-3**	1	12.2	**<0.001**	1	11.1	**1.22e-3**
	HYB	1	117	**<0.001**	-	-	-	-	-	-
	WGD×HYB	1	22.1	**<0.001**	-	-	-	-	-	-
	Residuals	184	-	-	84	-	-	100	-	-

*Proportion of TEs in transcriptomes (number of reads mapped to TEs and genes) was analyzed by generalized linear models (GLMs) with a quasi-binomial error distribution and a logit link function. WGD (diploid/tetraploid), hybridization (hybrid/non-hybrid) and batch/lane effect (lane ID) were added as categorical explanatory variables, assuming an interaction between WGD and hybridization. In all models, the effects of variables were tested with F-tests. Significant effects (p-value < 0.05) were highlighted in bold. A dash (“-”) indicates that the field is not applicable.

Methods S1 Ovule rescue

The flower buds of the diploid or tetraploid *C. orientalis* were emasculated. Two days after emasculation, the flowers were pollinated with pollen from diploid or tetraploid *C. grandiflora*, respectively. Four days after pollination, the ovules were dissected from the enlarged ovaries and were cultured on Murashige and Skoog (MS) medium with 8% (w/v) sucrose for 10 days (16-h light at 22°C and 8-h dark at 20°C, light intensity =137 $uE \cdot m^{-2} \cdot s^{-1}$). Later the ovules were transferred to MS medium with 1% (w/v) sucrose and cultured for one more month under the same conditions before being transplanted to soil.

Methods S2 Plant growing conditions

The seeds were first germinated in plastic Petri dishes with MS medium. After seven days of stratification at 4°C in the dark, the Petri dishes were moved to a growth chamber under long-day conditions (16-h light at 22°C and 8-h dark at 20°C, light intensity=137 $uE \cdot m^{-2} \cdot s^{-1}$). The day that the plants were moved into the growth chamber was regarded as germination start. Seven days after germination, the seedlings were transplanted to pots (8×8×6 cm) filled with soil and grown under the same photoperiodic and temperature conditions. The pots were distributed in 36 trays, and each tray contained eight individuals, including one from each of the seven groups.

The position of pots within each tray and the position of trays within the growth chamber were randomized.

Methods S3 Examining pollen viability and pollen counts

The viable and non-viable pollen grains were distinguished by staining pollen with aceto-carmine solution. Anthers were collected from the two oldest flower buds of each plant and air-dried in Eppendorf tubes with the lids open for five days at room temperature. The released pollen grains of each flower were stained and suspended in 30 µL acetic acid-carmine (5%, w/v)-TWEEN 20 (5%, v/v) solution (aceto-carmine solution), except Cg2 and Cg4 groups. To facilitate counting, pollen grains from Cg2 and Cg4 groups were suspended in 60 µL aceto-carmine solution. The viable and non-viable pollen grains were then counted under an optical microscope with a hemocytometer. Pollen grains staining pink were considered viable, and the ones that stayed transparent or greenish yellow were considered non-viable. The counts of five big counting chambers of the hemocytometer (0.5 µL in total) were used to calculate the number of pollen grains per flower (Pollen grains per flower = pollen counts * d, and the dilution factor d is 120 for Cg2 and Cg4 groups, and 60 for the rest of groups). At least 300 pollen grains per flower (>600 per individual) were inspected for measuring the proportion of viable pollen.

Methods S4 RNA extraction

Total RNA was extracted from leaves and inflorescences with a cetyl-trimethyl-ammonium-bromide (CTAB) based method. After the standard extraction with chloroform/isoamyl alcohol (24:1, v/v), total RNA was first precipitated with LiCl solution (final concentration 3 $mol \cdot L^{-1}$) for 2.5 hours at -20°C. Contaminating DNA in the RNA was further removed by an RNase-Free DNase Set (QIAGEN) with an in-solution method. The purified RNA was then precipitated again with isopropanol in presence of sodium acetate (final concentration 0.1 $mol \cdot L^{-1}$) for 30 minutes at -20°C. The concentration, purity and integrity of RNA were verified by agarose gel electrophoresis, a NanoDrop 2000 spectrophotometer (Thermo Scientific), and a Fragment Analyzer (Agilent Technologies).

Methods S5 Downsampling mapped read pairs

To mitigate the effect of sequencing depth variation, we downsampled the number of read pairs mapped to the reference genome with a custom python script, so that the average number of mapped read pairs (library size) was similar among the seven groups. Specifically, for individual i in group j, $L_{ij,\ downsampled} = L_{ij,\ original} *(\bar{L}_{min} / \bar{L}_{j})$, where L_{ij} is the library size of the individual i, $\bar{L}_{min}$ is the average library size of the group with the minimum library size, and $\bar{L}_{j}$ is the average library size of group j. Then $L_{ij,\ downsampled}$ mapped reads were randomly sampled from individual i, without replacement. The downsampling was performed for the two tissues separately, and the difference between the two tissues was not adjusted.
